# The many ways to inhibit translation by Sorafenib in liver cancer cells

**DOI:** 10.1007/s11010-025-05391-z

**Published:** 2025-09-15

**Authors:** Laura Contreras, Sara Ricciardi, Stefano Biffo, Jordi Muntané, Jesús de la Cruz

**Affiliations:** 1 Instituto de Biomedicina de Sevilla, Hospital Universitario Virgen del Rocío/CSIC/Universidad de Sevilla, 41013 Seville, Spain; 2 Departamento de Genética, Facultad de Biología, Universidad de Sevilla, Seville, Spain; 3 Departamento de Fisiología Médica y Biofísica, Universidad de Sevilla, Seville, Spain; 4 National Institute of Molecular Genetics, INGM Fondazione Romeo Ed Enrica Invernizzi, Milan, Italy; 5 Department of Biosciences, University of Milan, Milan, Italy; 6 Centro de Investigación Biomédica en Red de Enfermedades Hepáticas y Digestivas (CIBEREHD), Madrid, Spain

**Keywords:** Hepatocellular carcinoma, Sorafenib, eIF4E, ERK1/2 MAPK signalling, MNK1a, PERK

## Abstract

Sorafenib targets various tyrosine kinase receptors, inhibiting cell growth and proliferation, angiogenesis and metastasis in tumour cells. It is used to treat certain types of cancers including renal, thyroid and liver (hepatocellular carcinoma) cancers. Although Sorafenib is approved for advanced hepatocellular carcinoma, it only extends patient´s lives by a few months, highlighting the urgent need to better understand how it works and to develop more effective treatments. Sorafenib specifically inhibits translation initiation in hepatocellular carcinoma cells. Herein, we revealed that this inhibition results, at least, from the activation of PERK, triggering a stress response that leads to eIF2α phosphorylation, the inhibition of MNK1a-signalling-dependent eIF4E phosphorylation, and the aberrant assembly of the canonical eIF4F complex. Sorafenib also inhibits the ERK1/2 MAPK signalling in HepG2 cells. However, the mTORC1 pathway does appear to play a pivotal role in Sorafenib-dependent translation inhibition, as revealed by the phosphorylation levels of RPS6 and 4EBP1 proteins and the effects on translation of gene silencing 4EBP1/2 in Sorafenib-treated cells. Translation inhibition correlates with reduced production of cancer-promoting proteins like Cyclin D1 and c-Myc. Overexpression of the phosphomimetic eIF4E-S209D variant, which constitutively activates eIF4E, shows that inhibition of eIF4E phosphorylation directly causes Cyclin D1 down-regulation and cell-cycle delay in Sorafenib-treated cells. Taken together, our results confirm that Sorafenib induces translation reprogramming, whose understanding is crucial for improving its efficacy as a cancer therapy.

**Supplementary Information:**

The online version contains supplementary material available at 10.1007/s11010-025-05391-z.

## Introduction

Hepatocellular carcinoma (HCC) is one of the most common malignant tumours in adult males and females. HCC has been described as the sixth most common neoplasm and the fourth most frequent cause of cancer-related deaths in men and women worldwide [1, 2]. The occurrence and development of HCC is a complex issue involving a mixture of different aetiologies [3]. Most cases of HCC are associated with the presence of chronic liver disease due to hepatitis B virus (HBV) and hepatitis C virus (HCV) infection, diabetes, obesity, and metabolic-associated steatotic liver disease (MASLD). Additional risk factors that also promote HCC include toxins that act as carcinogens, such as tobacco smoke or food contaminants like aflatoxins [4].

HCC typically begins as an inflammatory process generating liver injury that progresses first to fibrosis and then to cirrhosis. The prognosis and clinical treatment of HCC vary depending on the staging of the tumour and the hepatic function of the patients. Among the different available classifications for HCC, the Barcelona Clinic Liver Classification (BCLC) staging system is currently one of the best methods to stage patients and recommend treatment. This classification is based on the size and number of tumour nodules in the liver, the presence of vascular invasion or extrahepatic metastasis, and the evaluation of the liver function and the general health status of the patient. Thus, while curative treatments such as local ablation, surgical resection, and orthotopic liver transplantation are indicated at very early and early stages of the disease, non-curative treatments such as chemoembolization, for example with cisplatin or doxorubicin, are the strategy at an intermediate stage as long as the portal artery has not invaded, the tumour nodules have not spread outside the liver, and the patients have a relatively preserved liver function [5]. At advanced stages, the treatments are systemic, including the administration, alone or in combination, of oral multi-tyrosine kinase inhibitors (mTKIs), such as Sorafenib (Sfb), Lenvatinib, Regorafenib, and Cabozantinib, and intravenous monoclonal antibodies against the programmed death-receptor ligand 1 (PD-L1; e.g., Atezolizumab or Durvalumab), the circulating vascular endothelial growth factor (VEGF; e.g., Bevacizumab), the VEGF receptor 2 (VEGFR-2; e.g., Ramucirumab), or the Cytotoxic T-Lymphocyte Antigen 4 (CTLA-4; e.g., Tremelimumab) [6, 7]. Currently, the combination of Atezolizumab with Bevacizumab is suggested as the first-choice of first-line treatment for patients in advanced stages of HCC, except for those for whom this treatment is not feasible, which could be treated with Sfb or Lenvatinib [7, 8]. Unfortunately, the overall benefit of these treatments, including that of Sfb, provides only very limited clinical benefit to patients, and although second- and third-line treatments are possible, including different oral mTKIs and monoclonal antibodies, there is need to develop novel and more effective therapies that, alone or in combination with mTKIs, could improve the outcomes for patients with advanced HCC [1]. In particular, elucidating the precise mechanism of Sfb action is essential to improve its anti-tumor efficacy and overcome its resistance [9].

At the molecular level, Sfb is an mTKI that can simultaneously inhibit VEGFR-2 and VEGFR-3, the platelet-derived growth factor receptor beta (PDGFR-ß), FLT3 and c-KIT, as well as the serine-threonine RAF kinases, which are integral components of the RAS/RAF/mitogen-activated protein (MAP)/extracellular signal-regulated kinase (ERK) kinase (MEK)/ERK signalling pathway [1, 10, 11]. Sfb exerts potent antiproliferative and pro-apoptotic activity against HCC cells and also triggers antiangiogenic effects [12]. Despite a clear correlation between the antitumor activity of Sfb and the inhibition of the MAPK signalling pathway (e.g., [13]), the precise molecular mechanisms by which Sfb exerts its clinical efficacy remain unclear. We and others have previously described that Sfb-induced apoptosis is related to the generation of endoplasmic reticulum (ER) stress in HCC and other cancer cells (e.g., [14, 15]). The Sfb-dependent ER stress response is linked to PKR-like ER kinase (PERK)-dependent activation to phosphorylate eIF2α, which leads to both protein synthesis inhibition through the initiation phase and induction of the unfolded protein response (UPR) [14–17]. As a consequence of stalling translation, Sfb has also been reported to promote the formation of stress granules [17]. However, despite the importance of Sfb interfering with protein synthesis, it is unclear how translation inhibition is initially triggered by this drug. The aim of the present study is to examine the different ways by which translation is targeted by Sfb in selected HCC cells. In this work, we show that Sfb inhibits translation through (i) the PERK-dependent induction of eIF2α phosphorylation, (ii) an ERK1/2 MAPK-signalling-dependent inhibition of phosphorylation of eIF4E through its downstream target MNK1a, and (iii) an aberrant assembly of the canonical eIF4F complex by reducing the proteins levels of its eIF4A and eIF4G components. Inhibition of translation correlates with a reprogramming involving at least the reduced production of critical proteins involved in oncogenesis, such as Cyclin D1 and c-Myc. Strikingly, herein we describe that the overexpression of an eIF4E phosphomimetic mutant (eIF4E-S209D) in Sfb-treated cells suppresses the down-regulation of Cyclin D1 protein levels, demonstrating that phosphorylation of eIF4E is directly involved in the expression regulation of this gene. Overexpression of either eIF4E-S209D or wild-type eIF4E also suppressed the cell-cycle delay induced by Sfb, which is unrelated to the function of Cyclin D1. We discuss on the importance of deciphering the Sfb-induced translation reprogramming to understand Sfb efficacy and develop novel and promising therapeutic strategies to improve its treatment outcome.

## Materials and methods

### Cell lines, culture conditions, and treatments

The hepatoblastoma HepG2 and HCC Huh7 cell lines were used in this study. The HepG2 cell line was obtained from the American Type Culture Collection (LGC Standards S.L.U., Barcelona, Spain). The Huh7 cell line was purchased from Apath (LLC, Brooklyn, NY, USA).

Cells were cultured in minimal essential medium (MEM) with Earle’s balanced salts and L-glutamine (ref. E15–825, PAA Laboratories Inc., Toronto, ON, Canada), supplemented with 10% fetal bovine serum (FBS, ref. F7524, MilliporeSigma, Burlington, MA, USA; Lot No. 022M3395, endotoxin < 0.2 EU/ml), 1% sodium pyruvate (ref. S11-003, PAA Laboratories Inc.), 1% non-essential amino acids (ref. M11-003, PAA Laboratories Inc.), and penicillin–streptomycin solution (100 U/ml-100µg/ml; ref. P11-010, PAA Laboratories Inc.). Cells were grown in culture flasks at 37 °C in a humidified incubator with 5% CO_2_ until reaching a density of 100,000 cells/cm^2^. The absence of mycoplasma contamination was routinely tested.

Different drugs were used in this study: (i) Sorafenib (Sfb, ref. FS10808, Carbosynth Ltd., Berkshire, UK) was dissolved in dimethyl sulfoxide (DMSO) as a 10 mM stock solution. (ii) Sirolimus (ref. 37,094, MilliporeSigma) was dissolved in DMSO as a 100 mM stock solution. (iii) The Mnk1 inhibitor 4-amino-5-(4-fluoroanilino)-pyrazolo [3, 4-d] pyrimidine or CGP57380 (ref. 454,861, MilliporeSigma) was dissolved in DMSO as a 20 mM stock solution. (iv) Cycloheximide (ref. C7698, MilliporeSigma) was dissolved in water as a 10 mg/ml stock solution. (v) Puromycin (ref. NP09203, Carbosynth Ltd., Compton, UK) was dissolved in water as a 10 mg/ml stock solution. All stock solutions were stored at – 20 °C. Treatments at the indicated concentrations (see Results) were performed 24 h after culture plating; lysates were obtained at different time points after the treatments.

### Polysome analysis and sucrose gradient fractionation

The protocol for polysome preparation has been previously described [14]. Some minor modifications were introduced. Briefly, cells were grown to 70% confluency in 100 mm dishes as described above and treated as indicated in each experiment. Typically, two dishes were used per condition assayed. Before harvesting the cells, 200 µg/ml cycloheximide was added and incubated for 5 min at 37 °C. Each dish was then placed on ice, the media aspirated, and the cultures washed twice with PBS without Ca^2+^ and Mg^2+^ containing 200 µg/ml cycloheximide. Then, 600 µl of ice-cold lysis buffer (10 mM Tris–HCl, pH 7.4, 150 mM NaCl, 10 mM MgCl_2_, 200 µg/ml cycloheximide, 2 mM DTT, 0.5% NP40) was added to one dish, cells were scraped, and transferred to the second dish. After scraping the cells from the second dish, the mixture was transferred to a 1.5 ml-Eppendorf tube. Tubes were incubated at 4 °C with gentle end-over-end rotation for 10 min and then centrifuged at 16,000 × *g* for 8 min at 4 °C in a refrigerated microfuge. The corresponding supernatants were recovered and the A_260_ measured using a NanoDrop ND-1000 Spectrophotometer (Thermo Fisher Scientific, Waltham, MA, USA). Eleven A_260_ units were loaded onto 10–50% (w/v) sucrose gradients, prepared in a buffer containing 50 mM Tris–acetate, pH 7.5, 50 mM NH_4_Cl, 12 mM MgCl_2_, and 1 mM DTT. The gradients were centrifuged at 260,110 × *g* (39,000 rpm) in a SW41 Ti rotor (Beckman Coulter Inc., Brea, CA, USA) for 2 h 45 min at 4 °C. Fractionation was performed with an ISCO UA-6 system (Teledyne ISCO Inc., Lincoln, NE, USA) equipped to continuously monitor the A_254_. When required, fractions of 1 ml were collected from the gradients and processed (see below).

### Protein extraction and western blot analysis

Protein extracts were obtained by lysing cell pellets at 100 °C for 10 min in 2X Laemmli buffer (125 mM Tris–HCl, pH 6.8, 4% SDS, 200 mM DTT, 0.02% bromophenol blue, 20% glycerol). Extracts were then sonicated in a Bioruptor (Diagenode, Seraing, Belgium) for 1 min at high intensity, and then subjected to 10% SDS-PAGE and transferred to nitrocellulose membranes (Amersham™ Protran® 0.45 µm, GE Healthcare, Chicago, IL, USA). Membranes were blocked for 1 h with 5% bovine serum albumin (BSA) in TTBS (15 mM Tris–HCl, pH 7.5, 200 mM NaCl, 0.1% (v/v) Tween-20), followed by incubation with primary antibodies overnight at 4 °C. The primary antibodies used in this study are listed in supplementary table S1. After washings with TTBS buffer, the membranes were incubated with horseradish peroxidase (HRP)-conjugated secondary antibodies (Bio-Rad Laboratories, Inc., Hercules, CA, USA) at a 1:5000 dilution at room temperature for 1 h. Proteins were detected using an enhanced chemiluminescence detection kit (Super-Signal™ West Pico, Thermo Fisher Scientific) in a ChemiDoc™ Touch Imaging System (Bio-Rad) and the relative intensity value quantified with the Image Lab software provided with this system.

### Puromycin labelling

The puromycin assay relies on the incorporation of puromycin into nascent polypeptides and its subsequent detection with a monoclonal antibody against the antibiotic. Approximately 8 × 10^5^ HepG2 cells were seeded in 6-well plates for each experimental condition. Puromycin (5 µg/ml) was added 10 min before harvesting the cells. Cells were collected, lysed, and proteins were analysed by western blotting as described above using an anti-puromycin antibody (**Supplemental** **Table S1**). The amount of puromycin incorporation was quantified by densitometry.

### Lentiviral shRNAs, cell transfection, and knockdown expression of PERK and 4E-BP1

The small hairpin RNA (shRNA) vector targeting human PERK was purchased from MilliporeSigma (Clone ID: TRCN0000262381). This vector is based on the Sigma/TRC MISSION pLKO.1 vector but modified to confer puromycin resistance. HepG2 cells were seeded in 96-well plates, reaching 80% confluency for transduction. Twenty-four hours later, cells were treated with polybrene (8 µg/ml) and transduced with lentiviral particles. The viral particles-containing medium was removed and replaced with fresh, pre-warmed complete culture medium. The next day, puromycin (2 µg/ml) was added for the selection of transduced cells. The appropriate concentration of puromycin was determined based on a previously performed kill curve experiment. Non-transduced control cells were also exposed to puromycin. Puromycin-containing medium was replaced every 3 days until the control cells died. Several puromycin-resistant clones were tested by western blot hybridisation to determine which one provided the optimal gene knockdown degree.

The shRNA vector targeting human 4E-BP1 and 4E-BP2 was created for this study. To do so, shRNA sequences were cloned into the PLKO.1 vector using *Age*I and *Eco*RI enzymes. Competent *Escherichia coli* cells were transformed and screened for positive clones. To confirm the identity of the shRNA construct, the candidates were sequenced. The sequences of both the shRNA and the primer used for sequencing are listed in **Supplemental Table S2**. The packaging of lentiviruses was done using Lenti-X 293 T cells. Cells were seeded in 100 mm cell culture dishes and transfected using GeneJuice with the envelope and packaging plasmids, pVSV- G and pCMV-dR8.91, respectively, along with the 4EBP1 and 4EBP2 shRNA constructs. Forty-eight hours later, the lentiviral particles-containing supernatant was collected and centrifuged at 67,114 × *g* in a Beckman SW41Ti rotor at 4 ºC for 2 h. The supernatant was discarded, the pellet resuspended in 100 µl of PBS buffer (137 mM NaCl, 27 mM KCl, 100 mM Na_2_HPO_4_, 18 mM KH_2_PO_4_, pH 7.4) and aliquoted for storage at − 80 ºC. HepG2 cells were infected as described above.

### Total RNA extraction and analysis

About 8 × 10^5^ HepG2 cells were seeded in 6-well plates for each experimental condition. Total RNA was extracted from each sample using a RNeasy mini kit according to the manufacturer's instructions (QIAGEN, Hilden, Germany). RNA was then stored at -80 ºC for further analyses. When required, 1 µg of RNA was treated with 1 µl of DNase I (Promega, Madison, WIS, USA) following the manufacturer's instructions. Finally, RT-PCR and qPCR were performed. To do so, RNA was first reverse transcribed using SuperScript™ III First Strand (Invitrogen, Thermo Fisher Scientific). Synthesis for RT-PCR was then done according to the manufacturer's instructions (Invitrogen, Thermo Fisher Scientific) and random hexamer primers (Hoffman-La Roche, Basel, Switzerland). RT-qPCR was performed using SYBR® Green Premix Ex Taq™ 2X (Takara Bio Inc., Kasutsu, Japan) and specific primers of each transcript. Primer pairs used for the RT-qPCR experiment are shown in **Supplemental Table S3**.

### RNA isolation from sucrose gradient fractions and analysis

Before polysome fractionation, 100µl of each extract was separated to extract RNA corresponding to the whole RNA sample in the profile. Upon polysome profiling, fractions of 1 ml were collected and pooled together according to the experimental design. Before RNA extraction, 0.5 µg of a commercial luciferase RNA (cat. L4561, Promega) in a volume of 2 µl was added to each pool to normalise for RNA recovery. A treatment with a proteinase K solution (37.5 µl 10% SDS, 7.5 µl 0.5 M EDTA and 4 µl 20 mg/ml proteinase K per 1 ml) was performed for 1 h at 50 °C. An equal volume of acidic phenol:chloroform:isoamyl alcohol (25:24:1, v/v) was added to each sucrose fraction pool, samples were mixed during 30 s and centrifuged for 10 min at maximum speed at 4 °C. Approximately 80% of the aqueous phase was place in a new tube, and an equal volume of chloroform was added. After mixing for 30 s using a vortex, samples were centrifuged 10 min at maximum speed at 4 °C. Again, 80% of the aqueous phase was placed in a new tube, and the RNA was precipitated using 1:10 of 3 M sodium acetate, pH 5.2, and 1.5 volumes of ethanol. The mixture was incubated overnight at − 20 °C. Then, samples were centrifuged at maximum speed for 30 min at 4 °C, the pellet was washed with 70% ethanol, and finally resuspended in RNase-free water. The RNA was then analysed by RT-qPCR as described above or stored at − 80 °C. Primer pairs used for the RT-qPCR experiment are shown in **Supplemental Table S3**. The data were processed by normalising to the whole RNA sample and the luciferase RNA used as external control. The percentage of mRNA was calculated, and the data were expressed as the mean ± the standard deviation (SD).

### Plasmid constructs and DNA transfection

The plasmid pLPCX-S209D-eIF4E (a gift from T. Aasen) has been previously reported [18]. The plasmid pLPCX-WT-eIF4E was generated by PCR-mediated mutagenesis using pLPCX-S209D-eIF4E as a template. The PCR setting was chosen according to the instructions of High-Fidelity DNA Polymerase (ref. M0515, New England Biolabs, Ipswich, MA, USA). The PCR product was sequenced to confirm the change of the mutant to the wild-type codon. The pair of primers used to generate the site-directed mutation were: eIF4E-WT-fw (5’CTACTAAGAGCGGCTCCACCACTAAAAATAG3’) and eIF4E-WT-rv (5’CTATTTTTAGTGGTGGAGCCGCTCTTAGTAG3’). The primers to sequence the final PCR product were: pLPCX-fw (5’AGCTCGTTTAGTGAACCGTCAGATC3’) and pLPCX-rv (5’ACCTACAGGTGGGGTCTTTCATTCCC3’). Other plasmids used in this study were pcDNA3-MNK1a-Flag and pcDNA3-MNK1b-Flag (gifts from E. Martín) [19].

Transfections were performed as follows: first, about 7 × 10^5^ HepG2 cells were seeded in 6-well plates for each experimental condition. Twenty-four hours later, cells were transfected with 2 µg of each plasmid using Lipofectamine™ 2000 (Invitrogen, Thermo Fisher Scientific) according to the manufacturer's instructions. Cell transfection was performed in serum-free medium in the absence of antibiotics. Six hours later, the Lipofectamine-containing medium was removed and replaced with fresh, pre-warmed complete culture medium. Further analyses were performed 48 h post-transfection.

### Cell cycle analysis

Cell cycle progression was assessed by flow cytometric analysis. For this, HepG2 cells were seeded in 6-well plates. Cells were harvested by trypsinisation and fixed overnight in 70% ethanol in PBS buffer at 4 °C. After this, cells were resuspended in PBS buffer and incubated with 0.5 mg/ml RNase A for 1 h at 37 °C. Propidium Iodine was added to a final concentration of 50 µg/ml and incubated for 20 min at room temperature. Finally, cells were filtered to avoid aggregation and subjected to flow cytometry analysis using a FACSCanto™ Flow Cytometer and the FACSDiva software (BD Biosciences, Franklin Lakes, NJ, USA).

### Reproducibility and statistical analyses

Experiments were conducted at least three times (independent biological replicates) with at least three technical replicates. In figures, only representative panels are shown. Numeric data are presented as the mean ± SD. Student’s *t*-test for paired or unpaired samples with confidence interval of 95% was used for estimation of statistical significance. Statistical analyses were performed with the Prism 6.01 software (Insight Partners, NY, USA). Significance between conditions was indicated with the symbols *p < 0.05, **p < 0.01, ***p < 0.001, and ****p < 0.0001.

## Results

### Sorafenib inhibits global translation

Cancer progression is habitually associated with aberrant mRNA translation activity, leading to an enhancement of global protein synthesis, increased translation of specific mRNAs encoding oncoproteins, and downregulation of tumour suppressor proteins [20–22]. This phenomenon, knows as translation reprogramming, allows cancer cells to adapt and survive under a variety of stressful conditions, grow and divide uncontrollably, trigger metastasis and resist anticancer therapy [23]. Thus, inhibition of the translation machinery is viewed as a promising target for cancer chemotherapy [24, 25].

Sfb, a mTKI used to treat kidney, liver and thyroid cancer [26], has potent antiproliferative, anti-angiogenic and pro-apoptotic properties that, unfortunately, poorly improve the survival rate of patients with advanced renal cell carcinoma (RCC) or HCC [9, 10]. In addition to serious adverse side effects, Sfb administration induces early occurrence of resistance, leading to treatment discontinuation [1, 27]. Different cellular pathways have been proposed to explain Sfb’s efficacy against cancer and resistance; however, the precise mechanisms by which Sfb exerts its antitumour activity and the exact causes that induce Sfb resistance remain unclear. We and others have found that Sfb is an inhibitor of translation at the level of the initiation phase at concentrations similar to or below those measured in the serum of Sfb-treated patients with HCC (*i.e.,* 10 µM) [28]. It has been reported that Sfb induces potent ER stress characterised by the activation of PERK-dependent phosphorylation of eIF2α at its S51 residue [14–16, 29], which is one of the best-described mechanisms known to inhibit translation initiation [20, 30, 31]. Moreover, other authors have shown that the effect of Sfb on protein synthesis could correlate with dephosphorylation of 4E-BP1 [16], which favours its inhibitory association with eIF4E [30, 31], and dephosphorylation of eIF4E [32], which appears to reduce nucleo-cytoplasmic mRNA transport and translation of a set of specific mRNAs [30, 33]. This work aims to better understand the molecular mechanisms underlying the specific translation inhibition exerted by Sfb and explain whether its anticancer properties can be explained, at least in part, by its impact on translation. To do so, we first treated HepG2 cells with two concentrations of Sfb (1 and 10 µM), and analysed the effects these treatments have on global translation by polysome profile analysis. Sfb was also used at a concentration of 10 µM during a 12-h time course study, based on previously published studies [14]. As a result, we found that Sfb efficiently blocks the formation of polysomes in a dose- and time-dependent manner (**Supplemental Fig. S1**). Inhibition of translation was essentially complete at 12 h after exposure to Sfb, as revealed by the drastic increase in the 80S peak and the concurrent decrease of ribosome engaged in translation, known as polysomes. We found that the effect of Sfb on polysome profiles is similar in different HCC cells, making it general and not specific to the particular HepG2 cell line (**Supplemental Fig. S2**).

**Fig. 1:**
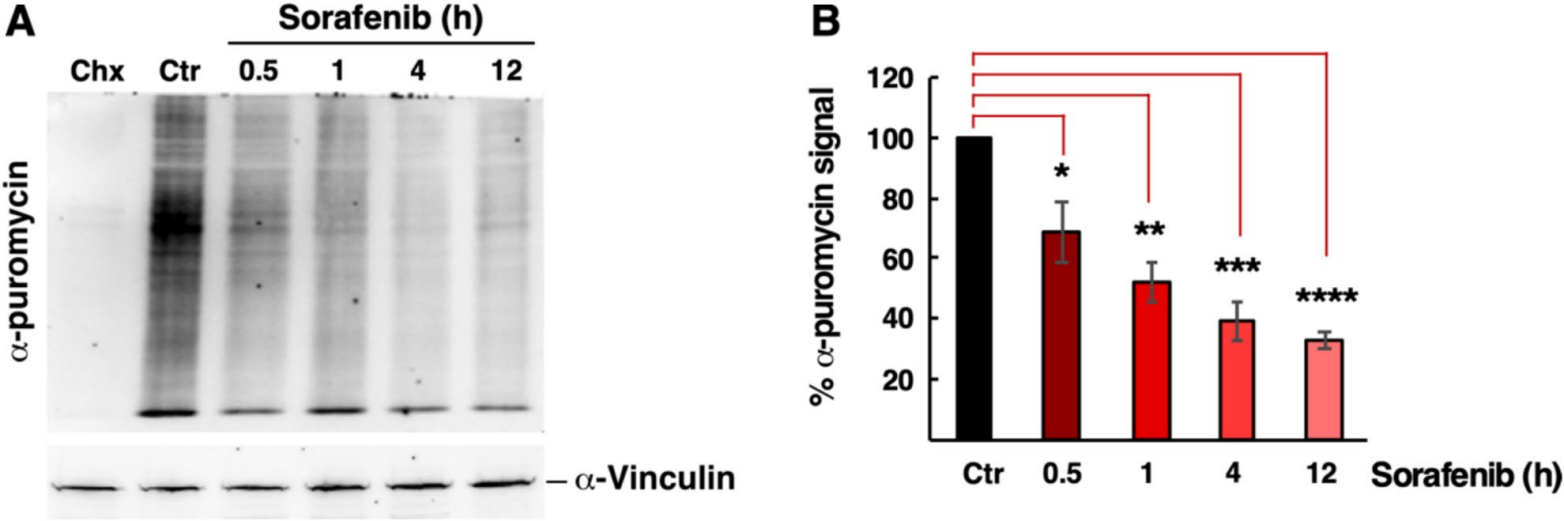
Time-course analysis of the translation inhibition exerted by Sorafenib using puromycin-labelled nascent proteins. **A** HepG2 cells were mock-treated (Ctr), treated with 100 µM cycloheximide for 30 min (Chx), or with 10 µM Sorafenib for the indicated times. Then, cells were exposed to 5 µg/ml puromycin for 10 min. Following protein extraction, puromycin incorporation into nascent proteins was detected by western blotting using a specific anti-puromycin antibody. The α-Vinculin protein was used as a loading control. A representative blot is shown. **B** Quantitation of puromycin incorporation was done by densitometric analysis. Data are presented as the mean of three independent experiments, and the error bars represent the SD. The control condition was taken as the reference value (100%) and the rest of samples normalized to it. Statistical significance was analyses by the Student's *t*-test (* *p* < 0.05; ** *p* < 0.01; *** *p* < 0.001; **** *p* < 0.0001)

To quantify the translation inhibition produced by Sfb, we performed a puromycilation assay, which measures the ability of cells to produce nascent proteins. To this end, we treated HepG2 cells with 10 µM Sfb to inhibit translation for 0.5, 1, 4 and 12 h, and then labelled nascent chains with puromycin. As expected, and in consonance with the polysome profile results, we observed a gradual reduction in puromycin-labelled polypeptides during the time course of Sfb treatment, with a significant reduction of about 30% after 30 min of exposure to Sfb and of more than 50% after 12 h (Fig. 1). We conclude that Sfb induces a rapid, dose- and time-dependent inhibition of global mRNA translation in HCC cells. These results complement our previous observations [14, 34] and are in agreement with earlier reports in different cancer cells [15–17, 32]. Whether this translation inhibition contributes to the anti-oncogenic impact of Sfb needs to be clarified.

### Contribution of the phosphorylation of eIF2α and 4E-BPs to the Sfb-induced translation inhibition

Two major mechanisms regulate translation initiation in mammalian cells: the increased phosphorylation status of eIF2α and the dephosphorylation of 4E-BPs [30]. Both mechanisms have been described to contribute to the translation inhibition exerted by Sfb (*e.g.* [14–17]*,*). We then assessed the phosphorylation status of eIF2α and 4E-BPs after a Sfb treatment in HepG2 cells by western blot hybridization using specific antibodies. As expected, both the phosphorylation levels of eIF2α at its Ser-51 residue and the phospho-eIF2α/eIF2α ratio significantly increased over time following Sfb treatment (**Supplemental Fig. S3A** and** B**). In contrast, both the phosphorylation levels of 4E-BP1 at its Ser-65 residue and the phospho-4E-BP1/4E-BP1 ratio remained unaltered after Sfb treatment for up to 4 h (**Supplemental Fig. S3C** and** D**). Similar results were found when analysing the phosphorylation levels of 4E-BP1 at its Thr-37 and Thr-46 residues (data not shown; see below Fig. 3). Thus, our results confirm that phosphorylation of eIF2α seems to play an important role in the translation inhibition exerted by Sfb, but suggest that the role of 4E-BP1, which is the major form of all cellular 4E-BPs, is apparently irrelevant in HepG2 cells upon Sfb treatment.

**Fig. 2:**
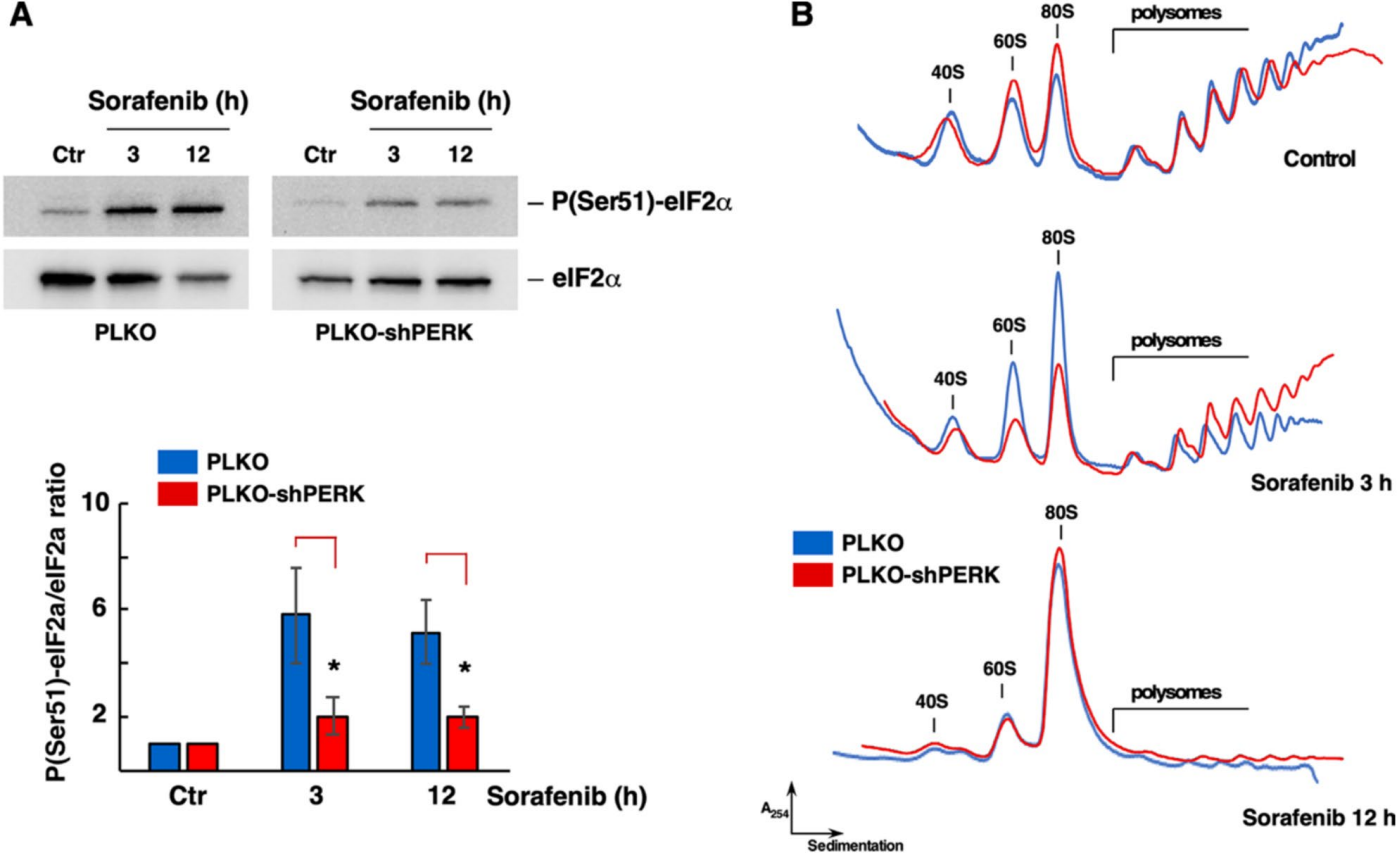
Silencing of PERK temporally restores the translation inhibition exerted by Sorafenib. **A** Effect on eIF2α phosphorylation upon silencing of PERK. Total protein extracts from PERK-silenced (PLKO-PERK) and control (PLKO) cells, mock-treated (Ctr) or treated with 10 µM Sorafenib for the indicated times, were analysed by western blotting with specific antibodies against the total and Ser51-phosphorylated forms of eIF2α. Upon densitometric analysis, the phospho-eIF2α/total eIF2α ratio was calculated and normalized to that of the control, which was set arbitrarily at 1.0 for each condition. Three independent experiments were performed; data are expressed as means ± SDs. Note that PERK silencing significantly reduces the phospho-eIF2α/eIF2α ratio. Statistical significance was analyses by the Student's *t*-test (* *p* < 0.05). **B** Translation is temporally restored in PERK-silenced cells. Translation was monitored by polysome profile analysis in the above cells and conditions. Ten A_260_ units of each extract were resolved in 7 to 50% sucrose gradients. The A_254_ was continuously monitored. Sedimentation is from left to right. The identity of the different peaks is indicated. Representative profiles are shown

To further define the precise role of these two factors in the translation inhibition induced by Sfb, we examined the translation status of Sfb-treated cells upon silencing the expression of either PERK, or 4E-BP1 and 4E-BP2. To do so, we created stable cell lines expressing shRNAs against the mRNAs encoding these proteins and, in parallel, we transfected control cells with an empty vector. We found a clear reduction in the levels of both eIF2α phosphorylation and total 4E-BP1/2 only after silencing the corresponding genes but not upon transfection with the empty vector (Fig. 2A and **Supplemental Fig. S4A**). Then, translation was analysed by polysome profile after the Sfb treatment. As a result, we found that Sfb induces a lower translation inhibition in PERK-silenced cells upon 3 h of treatment (Fig. 2B), indicating that PERK-induced phosphorylation of eIF2α plays a key role in the translation inhibition exerted by Sfb. Strikingly, this effect was not maintained in the long term, suggesting that other phospho-eIF2α-independent mechanisms must be involved over time in the Sfb translation inhibition. On the other hand, and in agreement with an irrelevant role of 4E-BPs in the translation inhibition exerted by Sfb in HepG2 cells, translation was not apparently ameliorated upon downregulation of 4E-BP1 and 4E-BP2 expression in the corresponding silenced cell line upon 3 h of Sfb treatment (**Supplemental Fig. S4B**).

### Sfb abrogates the phosphorylation of eIF4E

**Fig. 3:**
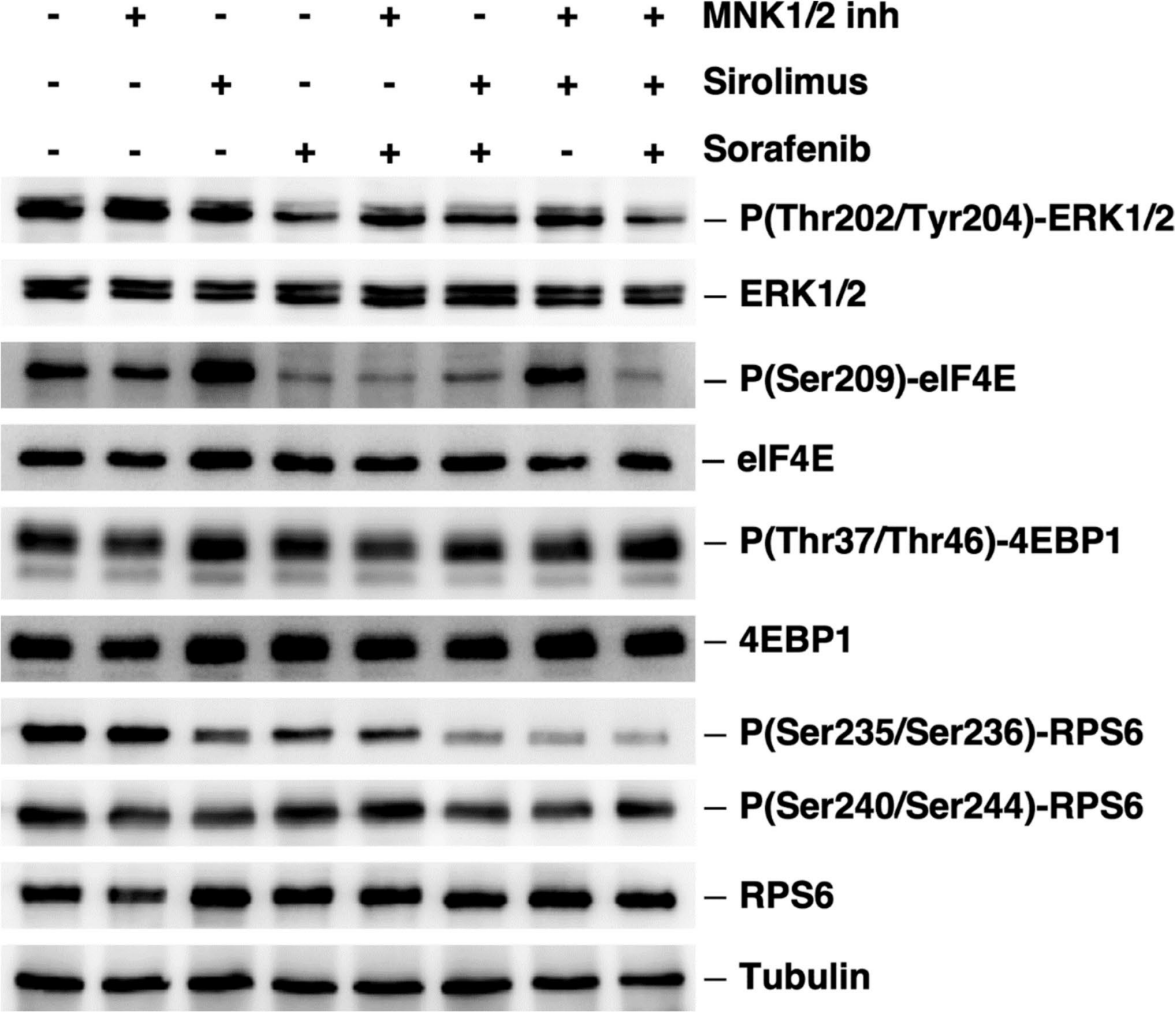
Sorafenib downregulates the MAPK signaling pathway and reduces the phospho-eIF4E levels. Total protein extracts from untreated or treated cells with 20 µM 4-amino-5-(4-fluoroanilino)- pyrazolo[3,4-d]pyrimidine (a MNK1/2 inhibitor), 100 nM Sirolimus (an mTORC1 inhibitor), and 10 µM Sorafenib for 1 h were obtained and analysed by western blotting as described in the Materials and Methods section. The absence of the drug is represented by a "-" mark. To test mTORC1 activity, the phospho-RPS6 and phospho-4E-BP1 protein levels were analysed using specific antibodies raised against P(Ser240/244)-RPS6, RPS6, P(Thr37/46)-4E-BP1 and 4E-BP1, respectively. To test the MAPKs pathway, phospho-ERK1/2 and phospho-eIF4E protein levels were analysed using specific antibodies raised against Thr202/Tyr204-ERK1/2, ERK1/2, P(Ser209)-eIF4E and eIF4E, respectively. Both pathways, mTORC1 and MAPKs, share the Ser235/236 of RPS6 as phosphorylation targets. Tubulin was used as a loading control. Representative blots are shown

Multiple signalling pathways converge on the translation machinery to regulate its function in response to a variety of extra- and intracellular stimuli, among them the Mitogen-activated protein kinases (MAPKs) and the mammalian Target of Rapamycin (mTOR) pathways [22, 35, 36]. Previously, it has been revealed that Sfb inhibits Akt, MEK and ERK phosphorylation (*e.g.* [13, 32, 37, 38]) and synergizes with mTOR inhibitors in different human cell lines, including HepG2 (*e.g.* [39–41]). To further evaluate the contribution of these pathways in the translation inhibition exerted by Sfb, we treated HepG2 cells for 1 h with the MAPK interacting kinase 1/2 (MNK1/2) inhibitor 4-amino-5-(4-fluoroanilino)-pyrazolo [3, 4-d] pyrimidine (also known as CGP57380) or the mTOR complex 1 (mTORC1) inhibitor Sirolimus and compared their impact on translation with that caused by Sfb by measuring the phosphorylation status of some of their specific targets by western blot hybridization (Fig. 3). Under basal conditions, and in agreement with an optimal translational rate, HepG2 cells displayed a high phosphorylation status of the MAPKs pathway components ERK1/2, the MNKs target eIF4E, and the mTORC1 targets 4E-BP1 and RPS6. Upon the MNK1 inhibitor treatment, and as expected [42–44], a mild dephosphorylation of eIF4E was observed. In contrast, the inhibition of mTORC1 with Sirolimus enhanced the phosphorylation levels of eIF4E, as previously reported [45, 46] and mildly reduced the phosphorylation status of RPS6, specially at its Ser-235 and Ser-236 [47]; however, the Sirolimus treatment seemed not enough to alter the phosphorylation status of 4E-BP1 at Thr-37 and Thr-46, which is in clear contrast to what has been previously reported (*e.g.* [48]). In turn, in our hands, Sfb strongly abrogated the phosphorylation of ERK1/2 and eIF4E and only mildly reduced that of RPS6 at Ser-235 and Ser-236. Altogether, these results suggest that, despite the induction on the phosphorylation of eIF2α, one of the early effects of Sfb on translation is the reduction in the phosphorylation status of eIF4E at its Ser-209, likely through the inhibition of the MAPK signalling pathway. In agreement with this, it has been reported that inactivation of mTORC1 results in a feedback activation of ERK1/2 [49], thus, the increase in the phosphorylation status of eIF4E that we experienced upon the Sirolimus treatment was completely abrogated when HepG2 cells were simultaneously treated with Sirolimus and Sfb (Fig. 3) and mildly reduced when simultaneously treated with Sirolimus and the MNK1 inhibitor CGP57380 (Fig. 3), in this latter case as previously reported [44]. Moreover, phosphorylation of RPS6 at Ser-235 and Ser-236 was also significantly reduced upon the Sfb treatment in combination with Sirolimus to levels similar to those reached when Sirolimus was combined with the MNK1 inhibitor (Fig. 3).

**Fig. 4:**
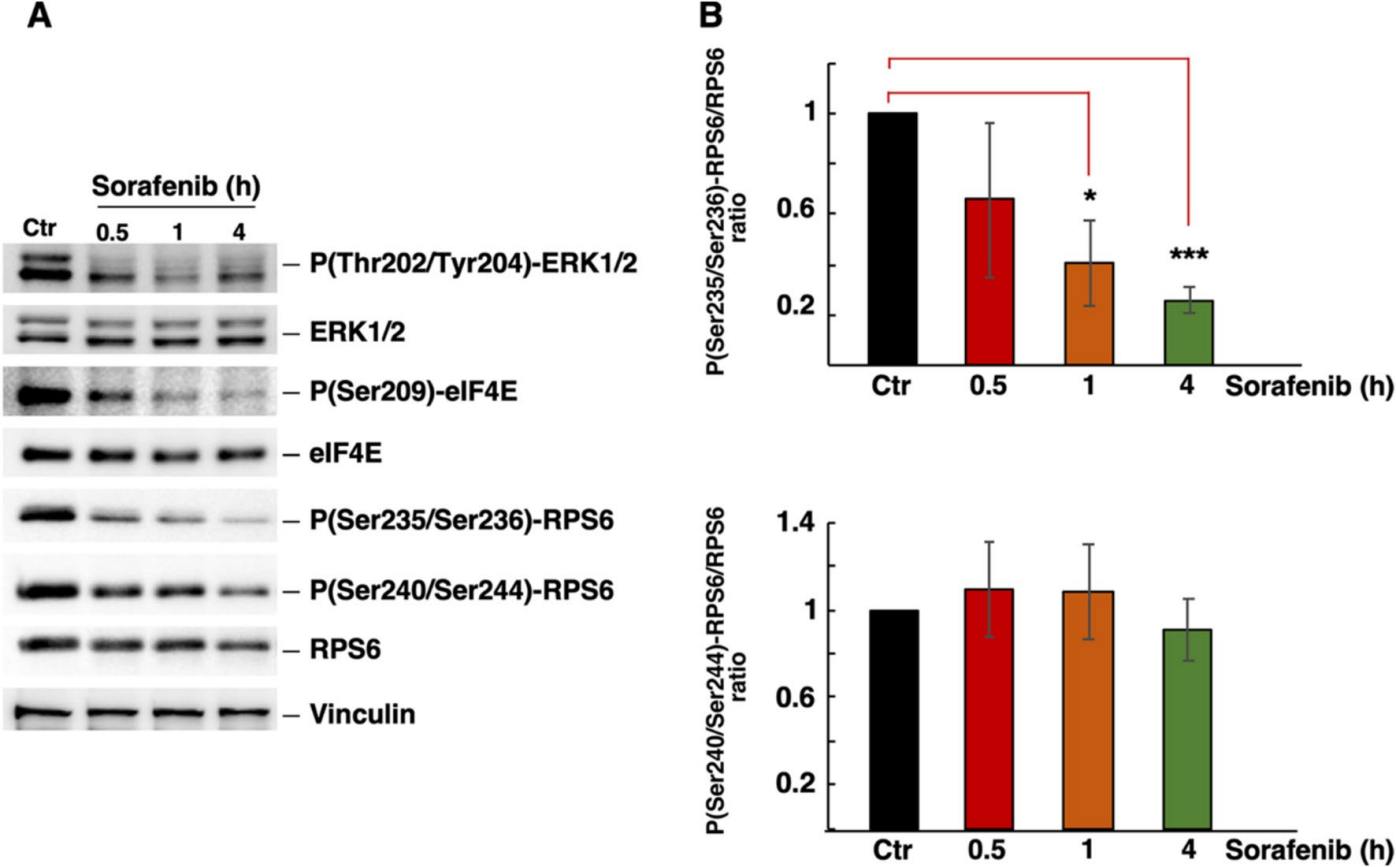
Sorafenib switches off MAPK signaling pathway leading to reduction in eIF4E phosphorylation. **A** Time-course of ERK1/2, eIF4E, and RPS6 phosphorylation. Total protein extracts from untreated (Ctr) or treated cells with 10 µM Sorafenib for the indicated times were obtained and analysed by western blotting as described in the Materials and Methods section. Vinculin was used as a loading control. The signals of total and phospho-ERK1/2 were detected using specific antibodies raised against ERK1/2 and P(Thr202/Tyr204)-ERK1/2, respectively. The signals of total and phospho-eIF4E were detected using specific antibodies raised against eIF4E and P(Ser209)-eIF4E, respectively. The signals of total and phospho-RPS6 were detected using specific antibodies raised against RPS6, P(Ser235/236)-RPS6 and P(Ser240/244)-RPS6. Representative blots are shown. **B** Densitometric analysis of the P(Ser235/236)-RPS6/RPS6 and P(Ser240/244)-RPS6/RPS6 ratios based on three independent replicates. Statistical significance was analysed by the Student's *t*-test (* *p* < 0.05; *** *p* < 0.001); Error bar: SD

To further analyse the effect of Sfb on the MAPKs pathway, we evaluated the phosphorylation levels of ERK1/2, eIF4E and RPS6 over a time course ranging from 0.5 to 4 h of drug treatment. Figure 4 shows a gradual reduction in the phosphorylation levels of eIF4E at Ser-209, ERK1/2 at Thr-202 and Tyr-204 and RPS6 at Ser-235 and Ser-236 with apparently similar kinetics. However, the phosphorylation of RPS6 at Ser-240 and Ser-244, which seems to be produced exclusively by mTORC1 [50], remained mostly unaffected by the Sfb treatment (see Fig. 4B, bottom panel). These data suggest that the MAPK pathway, rather than the mTORC1 pathway, plays a relevant role in the cellular response to Sfb in HepG2 cells. This is in agreement with our aforementioned results of silencing 4E-BPs (**Supplemental Fig. S4**) and in contrast to previously published studies [16].

### Translation of distinct eIF4E targets is reduced upon a Sfb treatment

**Fig. 5:**
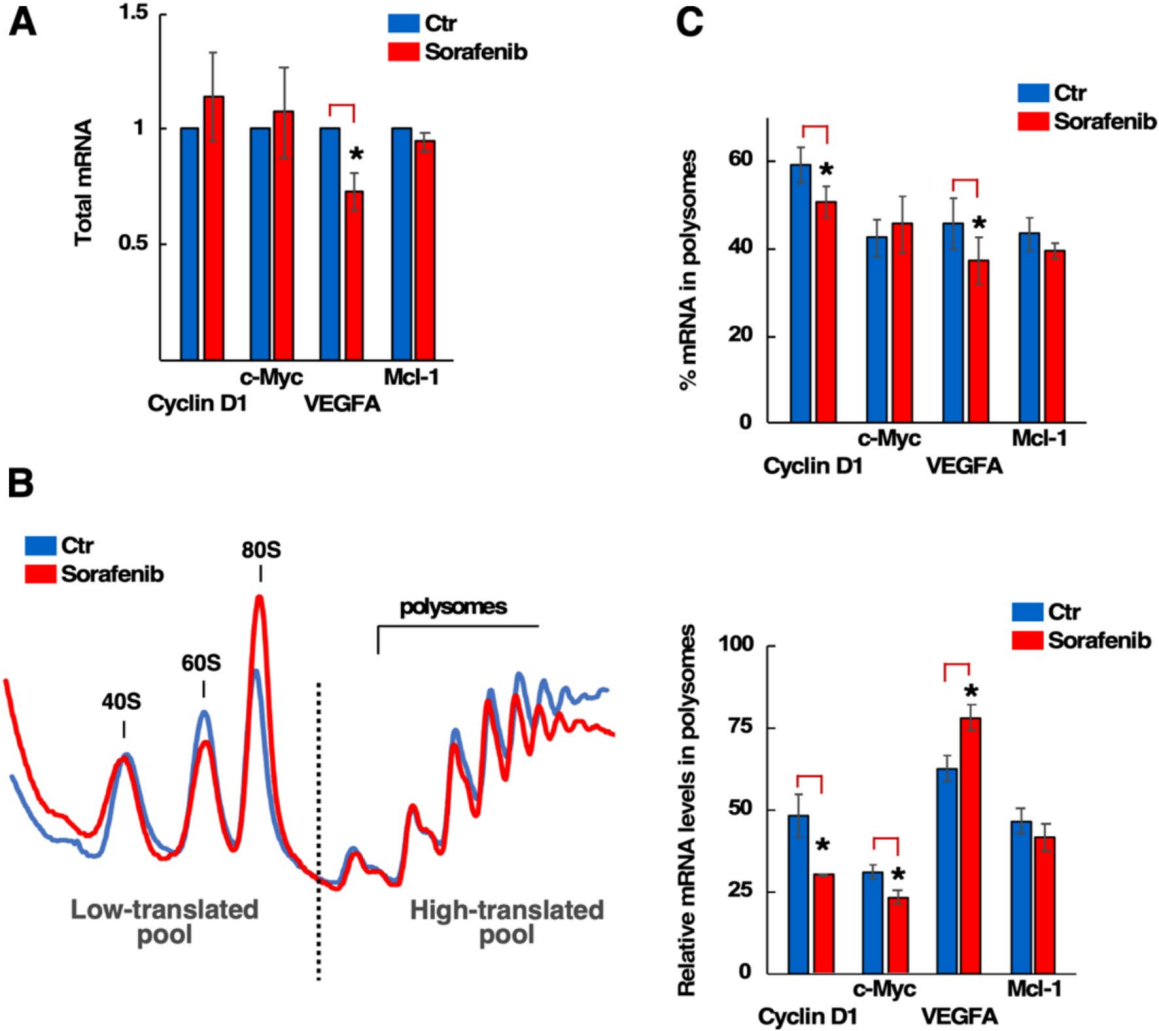
Translation of distinct eIF4E-sensitive mRNAs before or after Sorafenib treatment. **A** The mRNAs levels of four distinct eIF4E-sensitive genes (Cyclin D1, c-Myc, VEGFA, Mcl-1) were analysed by RT-PCR in total cellular RNAs extracted from HepG2 cells untreated (Ctr) or treated with 10 µM Sorafenib for 1 h. The mRNA levels were standardised against those of β-actin and normalized to the control condition. Analysis was based on four independent experiments. Statistical significances were determined using the Student’s *t*-test (* *p* < 0.05); Error bar: SD. **B** Polysome profile of cells treated with Sorafenib. Whole cell extracts from untreated or 10 µM Sorafenib-treated cells for 1 h were prepared and fractionated on sucrose gradients as described in the Materials and Methods section. Polysome profiles were recorded. Total RNA was isolated from the low-translated fraction (soluble fraction, free ribosomal subunits and monosomes) and the high-translated fraction (heavy polysomes). **C** Top panel: Histogram showing the percentage of mRNAs in polysomes. Bottom panel: Histogram showing the relative levels of mRNAs in polysomes. The levels of eIF4E-sensitive mRNAs were analysed by RT-PCR in total cellular RNA and RNA obtained from the fractions. Analysis was based on four independent experiments. Statistical significance was determined using Student’s *t*-test (* p < 0.05); Error bar, SD

**Fig. 6:**
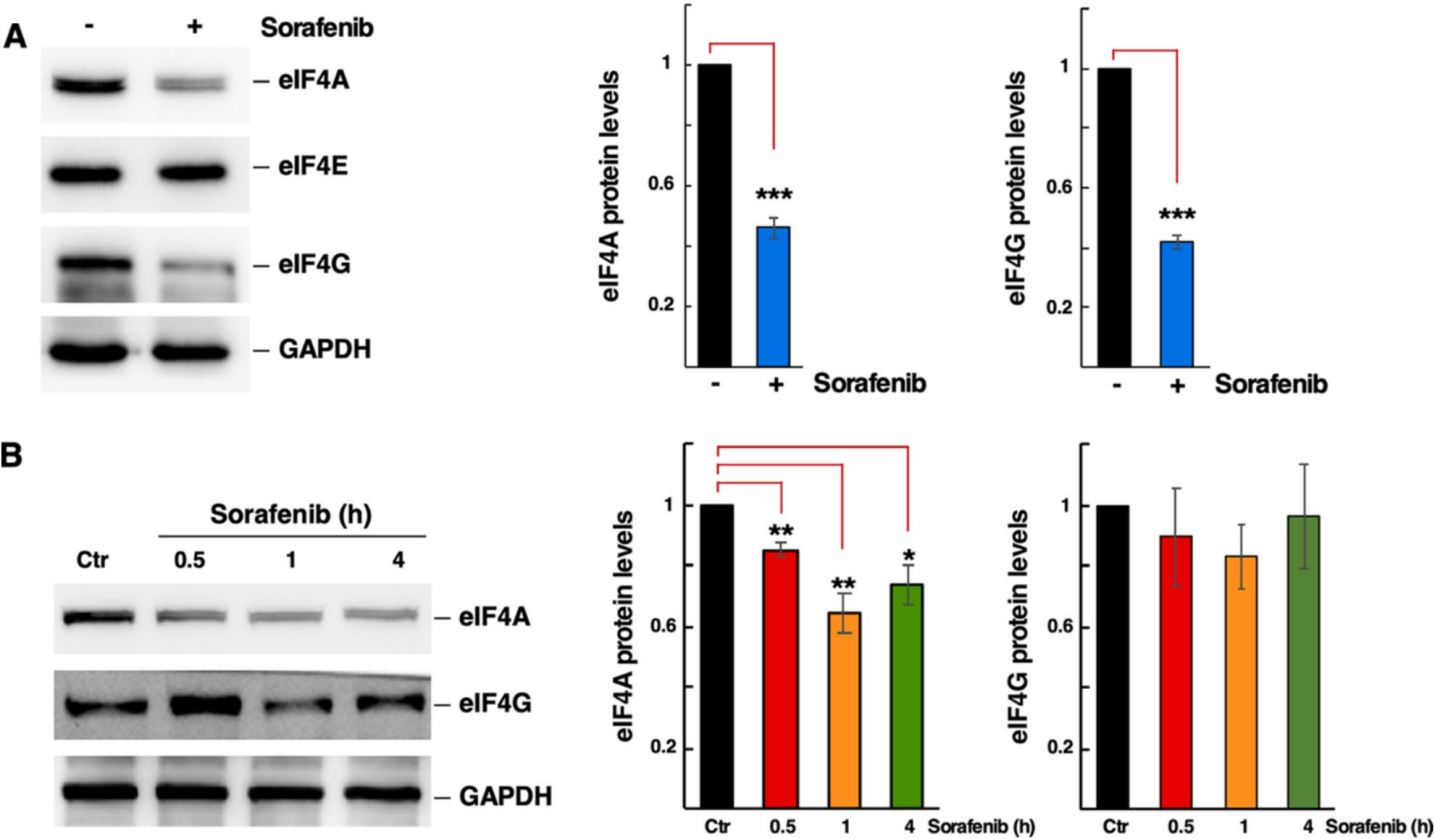
Levels of the cap-binding complex components upon Sorafenib treatment. (**A**) Total protein extracts from untreated (−) or treated (+) cells with 10 µM Sorafenib for 12 h were obtained and analysed by western blotting as described in the Materials and Methods section. GAPDH was used as a loading control. The signals of eIF4A, eIF4E, eIF4G, and GAPDH were detected using specific antibodies. Representative images are shown. The densitometric analysis based on three independent replicates is also shown. Statistical significance was determined using Student’s *t*-test (*** *p* < 0.001); Error bar: SD. **B** Protein levels of eIF4A and eIF4G after a Sorafenib time-course treatment. Total protein extracts from untreated (Ctr) or treated with 10 µM Sorafenib at the indicated times were obtained and analysed by western blotting as described in the Materials and Methods section. GAPDH was used as a loading control. The signals of eIF4A, eIF4G, and GAPDH were detected using specific antibodies. Representative images are shown. The densitometric analysis based on three independent replicates is also shown. Statistical significance was determined using Student’s *t*-test (* *p* < 0.05; ** *p* < 0.01); Error bar: SD

It is well established that eIF4E is the most limiting translation initiation factor, whose increased activity through overexpression and/or hyperphosphorylation enhances the translation of a subset of mRNAs (eIF4E-sensitive mRNAs) involved in oncogenesis. Among these are critical mRNAs encoding proteins required for cell proliferation, evasion of apoptosis, angiogenesis and metastasis, such as Cyclin D1, c-Myc, Mcl-1, and Vascular Endothelial Growth Factor A (VEGFA) [51–55]. Sfb reduces the phosphorylation of eIF4E; indeed, it has been hypothesised that Sfb-induced cell death is caused by a translational downregulation of Mcl-1 due to the inhibition of eIF4E phosphorylation [32, 56, 57]. Previously, we have also shown that levels of Mcl-1 decline upon long-term (16 h) Sfb treatment, as the earliest antiapoptotic protein downregulated in Sfb-treated HepG2 cells [14]. Here, we tested whether the translation status of distinct eIF4E targets changes after a short-term Sfb treatment in HepG2 cells. First, total RNA was isolated from untreated and Sfb-treated cells for 1 h, and the relative levels of mRNAs for the eIF4E targets Cyclin D1, c-Myc, Mcl-1 and VEGFA were analysed by RT-PCR (Fig. 5A). In parallel, polysome profiles were obtained, and fractions were combined into two pools, a low- and a high-translated one, from which RNA was also extracted and analysed by RT-PCR (Fig. 5B). The abundance of the above mRNAs in the fractions was assessed in two ways: (i) the percentage of mRNA, which compares the amount of mRNAs in the high-translated pool to that of the low-translated one, and (ii) the relative mRNA levels, which divides the amount of mRNA in the high-translated pool by its total cellular mRNA level. As shown in Fig. 5C, the percentage of the mRNAs of Cyclin D1 and VEGFA significantly decreased upon Sfb treatment, while those of Mcl-1 showed a slight, non-significant reduction. In addition, the relative mRNA levels of Cyclin D1 and c-Myc were significantly reduced by the Sfb treatment, while those of Mcl-1 had a non-significant tendency to decrease and those of apparently VEGFA increased, as the result of the reduction of its total mRNA levels in the samples of Sfb-treated cells (Fig. 5C; see also Fig. 5A). These effects seemed to be specific, as neither the percentage of mRNA nor the relative mRNA levels of distinct r-protein mRNAs (*RPS6*, *RPS18* and *RPL32*) significantly changed upon Sfb treatment (data not shown). To confirm that the translation of Cyclin D1 and c-Myc was down-regulated by Sfb in HepG2 cells, changes in the steady-state levels of these proteins were determined by western blot analyses after a time course of Sfb treatment from 0.5 to 4 h. As a result, a gradual reduction in Cyclin D1 and c-Myc proteins levels was detected. This reduction seemed to be specific to these eIF4E target proteins, as the levels of Tubulin, a protein selected as a control, remained constant during all points of the time course and similar to those detected in untreated cells (**Supplemental Fig. S5**). We decided not to explore Mcl-1 further, and unfortunately, we could not find an appropriate antibody against the VEGFA protein. Altogether, these results indicate that Sfb reduces the phosphorylation status of eIF4E at its Ser-209, which likely causes the specific translational downregulation of a subset of phospho-eIF4E targets. Moreover, it is tempting to suggest that the anti-proliferative, anti-angiogenic, and/or pro-apoptotic properties of Sfb could be partly explained as a result of this translation inhibition.

c-Myc regulates the expression of several components of the translation machinery, including the eIF4F complex components eIF4E, eIF4A and eIF4G. This establishes a positive feedforward loop, whereby c-Myc increases the levels of these translation factors, which in turn promote the translation of the c-Myc mRNA (*e.g.* [24, 58]). To investigate whether the Sfb treatment affects the protein levels of eIF4E, eIF4A and eIF4G, we determined their levels by western blot analyses. As shown in Fig. 6, both long-term (12 h) and short-term (up to 1 h) Sfb treatment caused an early reduction in eIF4A protein levels, followed by a later reduction in eIF4G; however, eIF4E levels remained apparently unchanged over time.

### Role of MNKs in the Sfb response

**Fig. 7:**
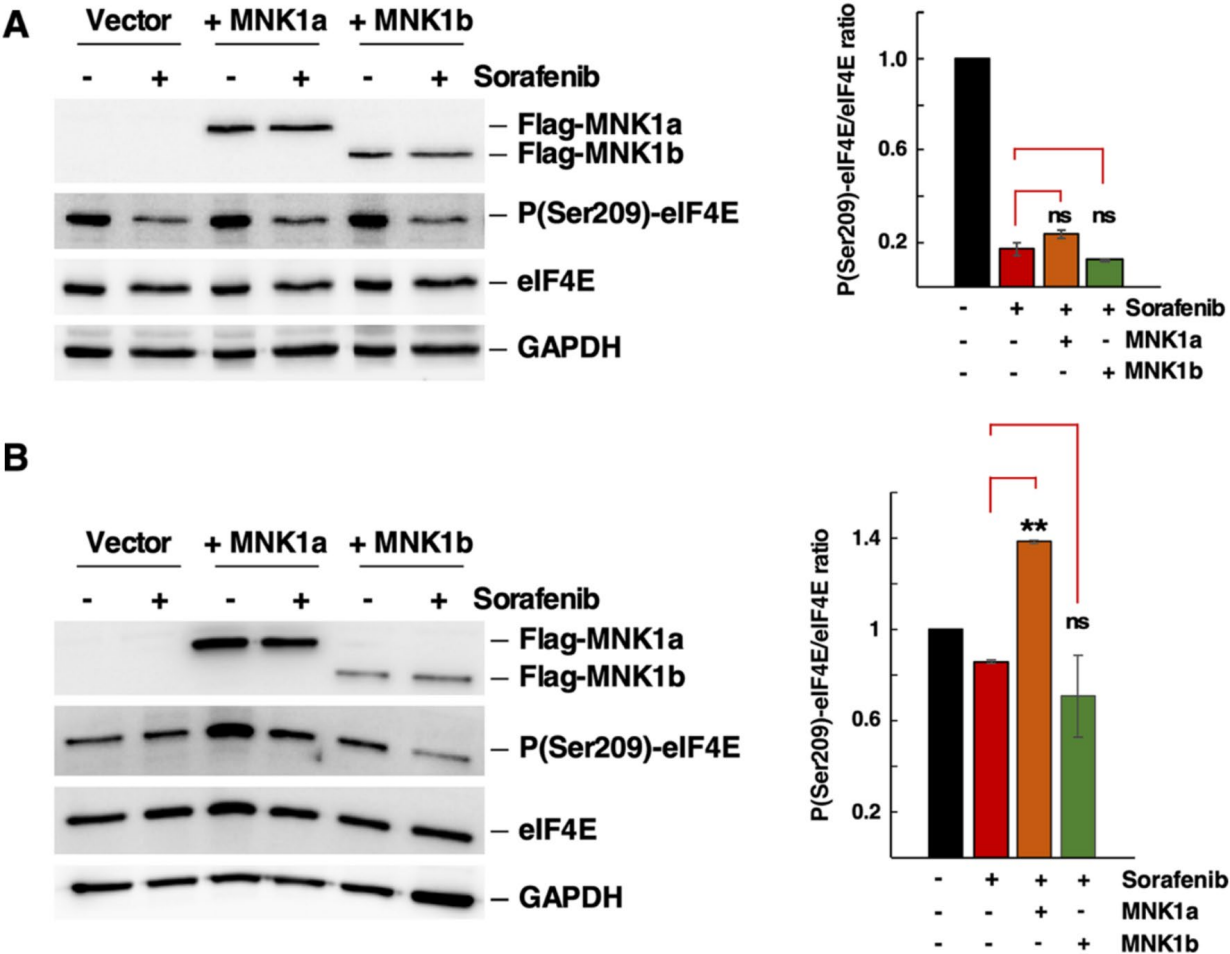
Role of MNK1 in the Sorafenib response. Cells were transfected with plasmids pcDNA3, pcDN3- MNK1a-Flag, and pcDNA3-MNK1b-Flag for 48 h. **A** Left panel: Total protein extracts from untreated (−) or treated (+) cells with 10 µM Sorafenib for 1 h were obtained and analysed by western blotting as described in the Materials and Methods section. The signals of exogenous MNK1a and MNK1b were detected using a specific antibody raised against the Flag epitope. Total and phospho-eIF4E were detected using eIF4E and P(Ser209)-eIF4E antibodies, respectively. GAPDH was used as a loading control. Right panel: The densitometric analysis of the phospho-eIF4E/eIF4E ratio based on three independent replicates is shown. Data were normalized to the control condition. Statistical significance was determined using Student’s *t*-test; Error bar, SD. ns, not significant. (**B**) Total protein extracts from untreated (−) or treated (+) cells with 1µM Sorafenib for 1 h were obtained and analysed by western blotting as described in the Materials and Methods section. The signals of exogenous MNK1a and MNK1b were detected using an antibody raised against the Flag epitope. Total and phospho-eIF4E were detected using eIF4E and P(Ser209)-eIF4E antibodies, respectively. GAPDH was used as loading control. Representative images are shown. Right panel: The densitometric analysis of the phospho-eIF4E/eIF4E ratio based on three independent replicates is shown. Statistical significance was determined using Student’s *t*-test (** *p* < 0.01); Error bar, SD. ns, not significant

The Ser-209 of eIF4E is the primary known substrate of MNK1 [59, 60], whose specificity requires its interaction with eIF4G [61, 62]. Two isoforms of MNK1 have been described in human cells: MNK1a and MNK1b. MNK1b is a spliced variant of MNK1a with distinct features in its C-terminal region [63, 64]. While MNK1b has high basal eIF4E-kinase activity that is poorly regulated by the MAPKs ERK and p38, the eIF4E-kinase activity of MNK1a is highly dependent on both MAPKs [64]. To better understand the role of MNK1 in response to Sfb, we overexpressed both MNK1a and MNK1b in HepG2 cells using pcDNA3-derived plasmids (see Materials and Methods) and evaluated the phosphorylation status of eIF4E after 12 h of Sfb treatment. As a result, neither MNK1a nor MNK1b overexpression significantly increased Ser-209 phosphorylation levels of eIF4E, which were reduced by the Sfb treatment, compared to control cells expressing endogenous levels of MNK1 (Fig. 7A). Therefore, overexpression of either MNK1 isoform is not enough to counteract the Sfb inhibition under the experimental conditions used. We then reanalysed the role of overexpression of MNK1 in cells treated with a lower dose of Sfb (1 µM) instead of the standard 10 µM. As a result, we observed that control cells showed a significant reduction of approximately 20% in eIF4E phosphorylation levels after this treatment (Fig. 7B). Interestingly, overexpression of MNK1a, but not that of MNK1b, was able to sustain Ser-209 phosphorylation of eIF4E (Fig. 7B). We conclude that MNK1a overexpression can counteract the negative effect of Sfb on the phosphorylation status of eIF4E only at low drug concentrations. However, this response is insufficient at the therapeutic concentration of 10 µM. Altogether, these results strongly suggest that the inhibitory effect of Sfb on the MAPK pathway is stronger than the positive suppression due to the MNK1 overexpression, leading to incomplete phosphorylation of eIF4E at its Ser-209.

### Role of the eIF4E phosphorylation in the Sfb-response

**Fig. 8:**
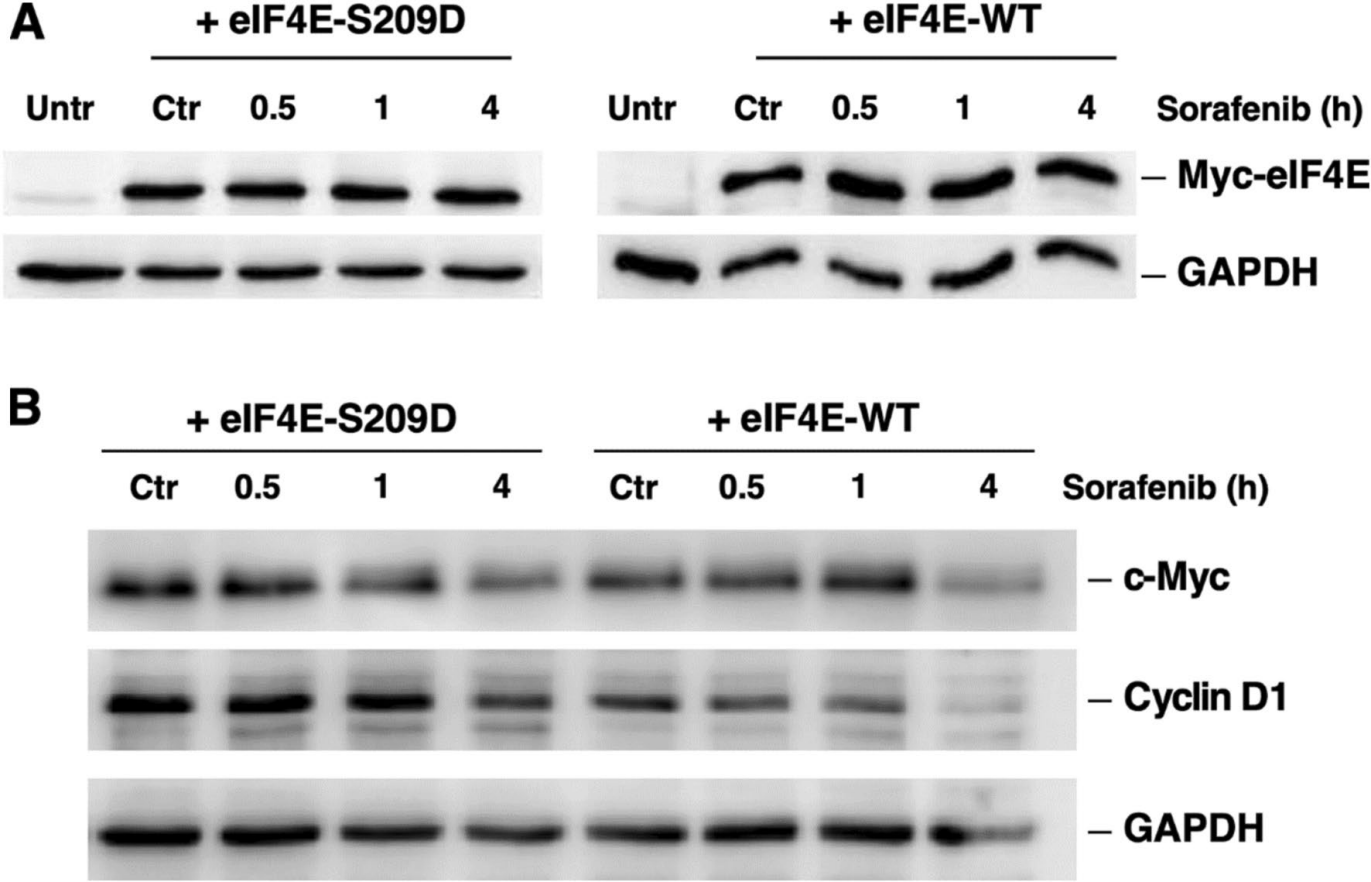
A phosphomimetic isoform of eIF4E partially restores the levels of distinct eIF4E-target proteins. **A** Total protein extracts from untransfected (Untr) cells and cells transfected with pLPCX-eIF4E-WT and pLPCX-eIF4E-S209D during 48 h, which were mock-treated (Ctr) or treated with 10 µM Sorafenib for the indicated times, were obtained and analysed by western blotting as described in the Materials and Methods section. The signals of exogenous Myc-eIF4E-WT and Myc-eIF4E-S209D were detected using a specific antibody raised against the Myc tag. GAPDH was used as a loading control. **B** The same total protein extracts from transfected cells were used to analyse the levels of c-Myc and Cyclin D1 proteins. The signals of Cyclin D1 and c-Myc were detected using specific antibodies. GAPDH was used as a loading control. Representative images are shown

To further explore the role of eIF4E phosphorylation in the Sfb response, we overexpressed either a wild-type (eIF4E-WT) or a phosphomimetic (eIF4E-S209D) isoform of eIF4E tagged with the Myc epitope from the pLPCX plasmid (see Materials and Methods). We then re-evaluated the protein levels of distinct phospho-eIF4E targets after 10 µM Sfb treatment for 0.5 to 4 h. As a result, cells overexpressing eIF4E-S209D showed practically unaltered Cyclin D1 levels compared to mock-treated cells overexpressing the eIF4E-WT isoform. However, the differences in c-Myc protein levels between cell overexpressing either isoform were not as clear. It appears that c-Myc levels were partially restored upon overexpression of either isoform compared with levels of non-transfected cells after 1 h of Sfb treatment (see Fig. 8 and **Supplemental Fig. S5**). Therefore, our findings strongly suggest that the downregulation of Cyclin D1 protein levels upon Sfb treatment is likely due to decreased eIF4E phosphorylation; however, this association does not apply to c-Myc protein levels, likely because Sfb dysregulates other cellular mechanisms controlling the expression of this factor.

Since Cyclin D1 is required for progression through the G1/S phase of the cell cycle, we analysed the cell cycle status of Sfb-treated cells overexpressing either eIF4E-WT or eIF4E-S209D by flow cytometry and compared it with that of control cells expressing endogenous levels of eIF4E-WT, that we have previously reported [34]. When treated with Sfb, control cells did not show a cell cycle arrest at G1; instead, they exhibited a significant but mild increase in the S-phase, which is accompanied with a reduction in G2 phase (**Supplemental Fig. S6**). This behaviour is similar to that observed in distinct thyroid cancer cells upon Sfb treatment [65]. Strikingly, this cell cycle arrest is suppressed upon overexpression of either eIE4E-WT or eIF4E-S209D (**Supplemental Fig. S6**). Taken together, these results suggest that phosphorylation of eIF4E at its Ser-209 mediates the cell cycle arrest induced by Sfb in HepG2 cells, but in a Cyclin D1-independent manner. Thus, eIF4E might play a more complex role in the Sfb response than initially suspected.

## Discussion

We and others have described the impact of Sfb on protein synthesis, which strongly and rapidly inhibits translation at the initiation phase in various HCC cell lines (this work and [14, 17, 34]). Translation initiation is the rate-limiting step in protein synthesis. In the present study, we have explored the contribution of different translation regulatory mechanisms in the Sfb response. We have confirmed and concluded that at least the phosphorylation of eIF2α, the phosphorylation of eIF4E, and the apparent aberrant assembly of the eIF4F complex are involved in modulating mRNA translation in response to Sfb. We focused on the HepG2 cell line, which has been extensively used to investigate a wide range of studies on liver cancer, including the cytotoxicity of Sfb. Moreover, we obtained similar data, including translation inhibition by Sfb and validation of the changes in protein levels of eIF4E (total and phosphorylation forms), eIF2 α(total and phosphorylation forms), eIF4G, c-Myc and Cyclin D1 upon a Sfb treatment, using other HCC lines such as Huh7 and SNU423 (**Supplemental** **Fig**. **S2** and data not shown).

Our results suggest that PERK plays a prominent role in the eIF2 α phosphorylation-mediated inhibition of translation in the short term of Sfb treatment. However, this inhibition is prolonged over time in an apparently PERK-independent manner. Consistent with elevated levels of phospho-eIF2α,we and others have found a significant enrichment of mRNAs with upstream open reading frames (uORFs) among the mRNAs that are differentially translated upon a Sfb treatment, including *ATF4*, which encodes a master transcription factor during the integrated stress response [17, 66, 67].

Our findings also highlight the role of the eIF4F complex in the Sfb regulation of translation. This consists of the cap-binding protein eIF4E, the scaffold protein eIF4G, and the RNA helicase eIF4A [68]. Specifically, we demonstrate that Sfb treatment inhibits eIF4A and eIF4G protein expression, which also have an impact in translation of sets of canonical cap-dependent and DAP5-dependent mRNAs (discussed in [67]).

In addition, Sfb rapidly inhibits eIF4E phosphorylation, especially when used at the therapeutic concentration of 10 µM, and mildly reduces eIF4E phosphorylation status at the lower concentration of 1µM. We have demonstrated that this reduction occurs as the consequence of targeting the RAS/RAF/MEK/ERK signalling pathway in HepG2 cells, as previously suggested in human leukaemia cells [57]. In contrast, phosphorylation of eIF4E-BP1 showed no major changes upon Sfb treatment. Indeed, despite the pivotal role of the PI3K/Akt/mTORC1 pathway in HCC (*e.g.* [69, 70]*,*), our results suggest that Sfb could inhibit translation in a manner not primarily involving this pathway. Thus, we have previously shown that high doses of mTOR inhibitors (Everolimus and Sirolimus) are required to inhibit translation, which strikingly and unexpectedly occurs at the early elongation rather than at the initiation phase [71]. Most importantly, in this report, we showed that the translation inhibition observed in HepG2 cells did not rely on the phosphorylation status or total levels of 4E-BP1. In this regard, a stable cell line with reduced expression of 4E-BP1 and 4E-BP2 upon gene silencing displayed an apparently similar degree of translation inhibition as control cells upon Sfb treatment. Finally, the fact that we could not detect extensive alterations in Sfb-treated cells in the phosphorylation status of RPS6 at its Ser-240 and Ser-244, a classical readout of mTORC1, further supported the observation that the activity of mTORC1 was not significantly affected by Sfb under our experimental conditions.

The eIF4E factor is known to be specifically phosphorylated at its Ser-209 by the MAP kinase-interacting kinases MNK1 and MNK2, which lie downstream of ERK1 and p38 MAP kinases [60, 72]. Interestingly, overexpression of either MNK1a or MNK1b failed to prevent the Sfb-mediated reduction of eIF4E phosphorylation at the therapeutic concentration of Sfb (10 µM), however, overexpression of MNK1a was able to fully suppress the small reduction in eIF4E phosphorylation caused by a lower concentration of Sfb (1 µM), further emphasising the role of this particular variant of MNKs in counteracting the effect of Sfb on eIF4E phosphorylation.

The underlying mechanisms based on the selective translation by eIF4E remain unclear, and the biological significance of the phosphorylation eIF4E at Ser-209 is still debated [73–75]. However, the importance of eIF4E in modulating translation has been clearly assessed [76]. Thus, translation of a subset of mRNAs, termed “eIF4E-sensitive or weak mRNAs”, has been described as strictly dependent on eIF4E [77]. Phosphorylation of eIF4E increases the translation efficiency of this group of mRNAs, including those encoding pro-tumorigenic factors such as Cyclin D1, VEGFA and c-Myc (*e.g.* [33, 54]*,*), whose translation we have observed is reduced upon a Sfb treatment. We have further analysed the role of Sfb on the phosphorylation status of eIF4E by studying the implications in translation of overexpressing an eIF4E phosphomimetic mutant (eIF4E-S209D) in Sfb-treated cells. As a result, we showed that overexpression of this variant suppresses the downregulation of at least Cyclin D1 protein levels following a short-term treatment with Sfb (4 h), demonstrating that phosphorylation of eIF4E is directly involved in the expression of this gene. In contrast, we did not observe a clear suppression of c-Myc protein levels, likely due to its intricate regulation by other translation factors and/or expression-controlling mechanisms also affected by Sfb treatment. We have found that Sfb induces a cell cycle delay in HepG2 cells, which does not occur in the G1/S phase that is the main phase regulated by Cyclin D1 (this work and [34]). Strikingly. overexpression of either eIF4E-S209D or wild-type eIF4E suppressed the cell-cycle delay induced by Sfb, thus, suggesting that Sfb negatively affects the cell-cycle in an eIF4E-dependent but Cyclin D1-independent manner.

Taken together, we propose a model that highlights the relevance of the translation machinery in the anti-tumorigenic properties of Sfb (**Supplemental Fig. S7**). On one hand, Sfb impairs the phosphorylation of eIF4E, leading to the selective translation inhibition of pro-tumoral mRNAs like Cyclin D1. Subsequently, Sfb also negatively affects the accumulation of eIF4A and eIF4G, which disrupts the assembly of the eIF4F complex and likely compromises the translation of canonical cap-dependent genes. This global protein synthesis inhibition is further reinforced by PERK-induced phosphorylation of eIF2 α. Further investigations, including studies in other cell lines (*e.g.* those resistant to Sfb) and in vivo validation, are required to substantiate our findings and assess their potential clinical relevance. Regardless of the context, we firmly believe that elucidating the mechanisms underlying Sfb-induced translation reprogramming is crucial for understanding Sfb's efficacy and ultimately developing novel and promising strategies aimed at improving its treatment outcomes.

## Supplementary Information

Below is the link to the electronic supplementary material.

Supplementary file1 (DOCX 928 kb)

## Data Availability

No datasets were generated or analysed during the current study.

## References

[1] Llovet JM, Kelley RK, Villanueva A, Singal AG, Pikarsky E, Roayaie S, Lencioni R, Koike K, Zucman-Rossi J, Finn RS (2021) Hepatocellular carcinoma. Nat Rev Dis Primers 7(1):6 33479224 10.1038/s41572-020-00240-3 PMC13537433

[2] El-Serag HB, Rudolph KL (2007) Hepatocellular carcinoma: epidemiology and molecular carcinogenesis. Gastroenterology 132(7):2557–2576 17570226 10.1053/j.gastro.2007.04.061

[3] Yang JD, Hainaut P, Gores GJ, Amadou A, Plymoth A, Roberts LR (2019) A global view of hepatocellular carcinoma: trends, risk, prevention and management. Nat Rev Gastroenterol Hepatol 16(10):589–604 31439937 10.1038/s41575-019-0186-y PMC6813818

[4] Lampimukhi M, Qassim T, Venu R, Pakhala N, Mylavarapu S, Perera T, Sathar BS, Nair A (2023) A review of incidence and related risk factors in the development of hepatocellular carcinoma. Cureus 15(11):e49429 38149129 10.7759/cureus.49429 PMC10750138

[5] Reig M, Forner A, Rimola J, Ferrer-Fabrega J, Burrel M, Garcia-Criado A, Kelley RK, Galle PR, Mazzaferro V, Salem R, Sangro B, Singal AG, Vogel A, Fuster J, Ayuso C, Bruix J (2022) BCLC strategy for prognosis prediction and treatment recommendation: the 2022 update. J Hepatol 76(3):681–693 34801630 10.1016/j.jhep.2021.11.018 PMC8866082

[6] Llovet JM, Castet F, Heikenwalder M, Maini MK, Mazzaferro V, Pinato DJ, Pikarsky E, Zhu AX, Finn RS (2022) Immunotherapies for hepatocellular carcinoma. Nat Rev Clin Oncol 19(3):151–172 34764464 10.1038/s41571-021-00573-2

[7] Mou L, Tian X, Zhou B, Zhan Y, Chen J, Lu Y, Deng J, Deng Y, Wu Z, Li Q, Song Y, Zhang H, Chen J, Tian K, Ni Y, Pu Z (2021) Improving outcomes of tyrosine kinase inhibitors in hepatocellular carcinoma: new data and ongoing trials. Front Oncol 11:752725 34707994 10.3389/fonc.2021.752725 PMC8543014

[8] Gordan JD, Kennedy EB, Abou-Alfa GK, Beal E, Finn RS, Gade TP, Goff L, Gupta S, Guy J, Hoang HT, Iyer R, Jaiyesimi I, Jhawer M, Karippot A, Kaseb AO, Kelley RK, Kortmansky J, Leaf A, Remak WM, Sohal DPS et al (2024) Systemic therapy for advanced hepatocellular carcinoma: ASCO guideline update. J Clin Oncol 42(15):1830–1850 38502889 10.1200/JCO.23.02745

[9] Fan G, Wei X, Xu X (2020) Is the era of sorafenib over? A review of the literature. Ther Adv Med Oncol 12:1758835920927602 32518599 10.1177/1758835920927602 PMC7252361

[10] Molina-Ruiz FJ, González R, Rodríguez-Hernández MA, Navarro-Villarán E, Padillo FJ, Muntané J (2016) Antitumoral activity of Sorafenib in hepatocellular carcinoma: effects on cell survival and death pathways, cell metabolism reprogramming, and nitrosative and oxidative stress. Crit Rev Oncog 21(5–6):413–432 29431086 10.1615/CritRevOncog.2017021302

[11] Cervello M, Bachvarov D, Lampiasi N, Cusimano A, Azzolina A, McCubrey JA, Montalto G (2012) Molecular mechanisms of sorafenib action in liver cancer cells. Cell Cycle 11(15):2843–2855 22801548 10.4161/cc.21193

[12] Llovet JM, Ricci S, Mazzaferro V, Hilgard P, Gane E, Blanc JF, de Oliveira AC, Santoro A, Raoul JL, Forner A, Schwartz M, Porta C, Zeuzem S, Bolondi L, Greten TF, Galle PR, Seitz JF, Borbath I, Haussinger D, Giannaris T et al (2008) Sorafenib in advanced hepatocellular carcinoma. N Engl J Med 359(4):378–390 18650514 10.1056/NEJMoa0708857

[13] Wilhelm SM, Carter C, Tang L, Wilkie D, McNabola A, Rong H, Chen C, Zhang X, Vincent P, McHugh M, Cao Y, Shujath J, Gawlak S, Eveleigh D, Rowley B, Liu L, Adnane L, Lynch M, Auclair D, Taylor I et al (2004) BAY 43–9006 exhibits broad spectrum oral antitumor activity and targets the RAF/MEK/ERK pathway and receptor tyrosine kinases involved in tumor progression and angiogenesis. Cancer Res 64(19):7099–7109 15466206 10.1158/0008-5472.CAN-04-1443

[14] Rodríguez-Hernández MA, González R, de la Rosa AJ, Gallego P, Ordóñez R, Navarro-Villarán E, Contreras L, Rodríguez-Arribas M, González-Gallego J, Álamo-Martínez JM, Marín-Gómez LM, del Campo JA, Quiles JL, Fuentes JM, de la Cruz J, Mauriz JL, Padillo FJ, Muntané J (2018) Molecular characterization of autophagic and apoptotic signaling induced by sorafenib in liver cancer cells. J Cell Physiol 234(1):692–708 30132846 10.1002/jcp.26855

[15] Rahmani M, Davis EM, Crabtree TR, Habibi JR, Nguyen TK, Dent P, Grant S (2007) The kinase inhibitor sorafenib induces cell death through a process involving induction of endoplasmic reticulum stress. Mol Cell Biol 27(15):5499–5513 17548474 10.1128/MCB.01080-06 PMC1952105

[16] Sauzay C, Louandre C, Bodeau S, Anglade F, Godin C, Saidak Z, Fontaine JX, Usureau C, Martin N, Molinie R, Pascal J, Mesnard F, Pluquet O, Galmiche A (2018) Protein biosynthesis, a target of sorafenib, interferes with the unfolded protein response (UPR) and ferroptosis in hepatocellular carcinoma cells. Oncotarget 9(9):8400–8414 29492203 10.18632/oncotarget.23843 PMC5823558

[17] Adjibade P, St-Sauveur VG, Quevillon Huberdeau M, Fournier MJ, Savard A, Coudert L, Khandjian EW, Mazroui R (2015) Sorafenib, a multikinase inhibitor, induces formation of stress granules in hepatocarcinoma cells. Oncotarget 6(41):43927–43943 26556863 10.18632/oncotarget.5980 PMC4791277

[18] Martínez A, Sesé M, Losa JH, Robichaud N, Sonenberg N, Aasen T, RyC S (2015) Phosphorylation of eIF4E confers resistance to cellular stress and DNA-damaging agents through an Interaction with 4E-T: a rationale for novel therapeutic approaches. PLoS ONE 10(4):e0123352 25923732 10.1371/journal.pone.0123352 PMC4414544

[19] Pinto-Díez C, García-Recio EM, Pérez-Morgado MI, García-Hernández M, Sanz-Criado L, Sacristán S, Toledo-Lobo MV, Pérez-Mies B, Esteban-Rodríguez I, Pascual A, Garcia-Villanueva M, Martínez-Jañez N, González VM, Martín ME (2018) Increased expression of MNK1b, the spliced isoform of MNK1, predicts poor prognosis and is associated with triple-negative breast cancer. Oncotarget 9(17):13501–13516 29568373 10.18632/oncotarget.24417 PMC5862594

[20] Robichaud N, Sonenberg N, Ruggero D, Schneider RJ (2019) Translational control in cancer. Cold Spring Harb Perspect Biol 11(7):a032896 29959193 10.1101/cshperspect.a032896 PMC6601465

[21] Lee LJ, Papadopoli D, Jewer M, Del Rincon S, Topisirovic I, Lawrence MG, Postovit LM (2021) Cancer plasticity: the role of mRNA translation. Trends Cancer 7(2):134–145 33067172 10.1016/j.trecan.2020.09.005 PMC8023421

[22] Chu J, Cargnello M, Topisirovic I, Pelletier J (2016) Translation initiation factors: reprogramming protein synthesis in cancer. Trends Cell Biol 26(12):918–933 27426745 10.1016/j.tcb.2016.06.005

[23] Fabbri L, Chakraborty A, Robert C, Vagner S (2021) The plasticity of mRNA translation during cancer progression and therapy resistance. Nat Rev Cancer 21(9):558–577 34341537 10.1038/s41568-021-00380-y

[24] Bhat M, Robichaud N, Hulea L, Sonenberg N, Pelletier J, Topisirovic I (2015) Targeting the translation machinery in cancer. Nat Rev Drug Discov 14(4):261–278 25743081 10.1038/nrd4505

[25] Kovalski JR, Kuzuoglu-Ozturk D, Ruggero D (2022) Protein synthesis control in cancer: selectivity and therapeutic targeting. EMBO J 41(8):e109823 35315941 10.15252/embj.2021109823 PMC9016353

[26] Wilhelm S, Carter C, Lynch M, Lowinger T, Dumas J, Smith RA, Schwartz B, Simantov R, Kelley S (2006) Discovery and development of sorafenib: a multikinase inhibitor for treating cancer. Nat Rev Drug Discov 5(10):835–844 17016424 10.1038/nrd2130

[27] Zhu YJ, Zheng B, Wang HY, Chen L (2017) New knowledge of the mechanisms of sorafenib resistance in liver cancer. Acta Pharmacol Sin 38(5):614–622 28344323 10.1038/aps.2017.5 PMC5457690

[28] Fucile C, Marenco S, Bazzica M, Zuccoli ML, Lantieri F, Robbiano L, Marini V, Di Gion P, Pieri G, Stura P, Martelli A, Savarino V, Mattioli F, Picciotto A (2015) Measurement of sorafenib plasma concentration by high-performance liquid chromatography in patients with advanced hepatocellular carcinoma: is it useful the application in clinical practice? A pilot study. Med Oncol 32(1):335 25429830 10.1007/s12032-014-0335-7

[29] Shi YH, Ding ZB, Zhou J, Hui B, Shi GM, Ke AW, Wang XY, Dai Z, Peng YF, Gu CY, Qiu SJ, Fan J (2011) Targeting autophagy enhances sorafenib lethality for hepatocellular carcinoma via ER stress-related apoptosis. Autophagy 7(10):1159–1172 21691147 10.4161/auto.7.10.16818

[30] Hershey JWB, Sonenberg N, Mathews MB (2019) Principles of translational control. Cold Spring Harb Perspect Biol 11(9):a032607 29959195 10.1101/cshperspect.a032607 PMC6719596

[31] Merrick WC, Pavitt GD (2018) Protein synthesis initiation in eukaryotic cells. Cold Spring Harb Perspect Biol 10(12):a033092 29735639 10.1101/cshperspect.a033092 PMC6280705

[32] Liu L, Cao Y, Chen C, Zhang X, McNabola A, Wilkie D, Wilhelm S, Lynch M, Carter C (2006) Sorafenib blocks the RAF/MEK/ERK pathway, inhibits tumor angiogenesis, and induces tumor cell apoptosis in hepatocellular carcinoma model PLC/PRF/5. Cancer Res 66(24):11851–11858 17178882 10.1158/0008-5472.CAN-06-1377

[33] Yang X, Zhong W, Cao R (2020) Phosphorylation of the mRNA cap-binding protein eIF4E and cancer. Cell Signal 73:109689 32535199 10.1016/j.cellsig.2020.109689 PMC8049097

[34] Contreras L, Rodríguez-Gil A, Muntané J, de la Cruz J (2022) Broad transcriptomic impact of Sorafenib and its relation to the antitumoral properties in liver cancer cells. Cancers (Basel). 10.3390/cancers14051204 35267509 10.3390/cancers14051204 PMC8909169

[35] Topisirovic I, Sonenberg N (2011) MRNA translation and energy metabolism in cancer: the role of the MAPK and mTORC1 pathways. Cold Spring Harb Symp Quant Biol 76:355–367 22123850 10.1101/sqb.2011.76.010785

[36] Mendoza MC, Er EE, Blenis J (2011) The Ras-ERK and PI3K-mTOR pathways: cross-talk and compensation. Trends Biochem Sci 36(6):320–328 21531565 10.1016/j.tibs.2011.03.006 PMC3112285

[37] Rosenberg L, Yoon CH, Sharma G, Bertagnolli MM, Cho NL (2018) Sorafenib inhibits proliferation and invasion in desmoid-derived cells by targeting Ras/MEK/ERK and PI3K/Akt/mTOR pathways. Carcinogenesis 39(5):681–688 29538717 10.1093/carcin/bgy038

[38] Wan PT, Garnett MJ, Roe SM, Lee S, Niculescu-Duvaz D, Good VM, Jones CM, Marshall CJ, Springer CJ, Barford D, Marais R, Cancer Genome P (2004) Mechanism of activation of the RAF-ERK signaling pathway by oncogenic mutations of B-RAF. Cell 116(6):855–867 15035987 10.1016/s0092-8674(04)00215-6

[39] Ramakrishnan V, Timm M, Haug JL, Kimlinger TK, Halling T, Wellik LE, Witzig TE, Rajkumar SV, Adjei AA, Kumar S (2012) Sorafenib, a multikinase inhibitor, is effective in vitro against non-Hodgkin lymphoma and synergizes with the mTOR inhibitor rapamycin. Am J Hematol 87(3):277–283 22190165 10.1002/ajh.22263 PMC3465673

[40] Yi H, Ye T, Ge M, Yang M, Zhang L, Jin S, Ye X, Long B, Li L (2018) Inhibition of autophagy enhances the targeted therapeutic effect of sorafenib in thyroid cancer. Oncol Rep 39(2):711–720 29207150 10.3892/or.2017.6118

[41] Gedaly R, Angulo P, Chen C, Creasy KT, Spear BT, Hundley J, Daily MF, Shah M, Evers BM (2012) The role of PI3K/mTOR inhibition in combination with sorafenib in hepatocellular carcinoma treatment. Anticancer Res 32(7):2531–2536 22753710

[42] Knauf U, Tschopp C, Gram H (2001) Negative regulation of protein translation by mitogen-activated protein kinase-interacting kinases 1 and 2. Mol Cell Biol 21(16):5500–5511 11463832 10.1128/MCB.21.16.5500-5511.2001 PMC87272

[43] Zhang Y, Li Y, Yang DQ (2008) Phosphorylation of eIF-4E positively regulates formation of the eIF-4F translation initiation complex following DNA damage. Biochem Biophys Res Commun 367(1):54–59 18164262 10.1016/j.bbrc.2007.12.118

[44] Huang XB, Yang CM, Han QM, Ye XJ, Lei W, Qian WB (2018) MNK1 inhibitor CGP57380 overcomes mTOR inhibitor-induced activation of eIF4E: the mechanism of synergic killing of human T-ALL cells. Acta Pharmacol Sin 39(12):1894–1901 30297804 10.1038/s41401-018-0161-0 PMC6289382

[45] Sun SY, Rosenberg LM, Wang X, Zhou Z, Yue P, Fu H, Khuri FR (2005) Activation of Akt and eIF4E survival pathways by rapamycin-mediated mammalian target of rapamycin inhibition. Cancer Res 65(16):7052–7058 16103051 10.1158/0008-5472.CAN-05-0917

[46] Wang X, Yue P, Chan CB, Ye K, Ueda T, Watanabe-Fukunaga R, Fukunaga R, Fu H, Khuri FR, Sun SY (2007) Inhibition of mammalian target of rapamycin induces phosphatidylinositol 3-kinase-dependent and Mnk-mediated eukaryotic translation initiation factor 4E phosphorylation. Mol Cell Biol 27(21):7405–7413 17724079 10.1128/MCB.00760-07 PMC2169067

[47] Liu L, Li F, Cardelli JA, Martin KA, Blenis J, Huang S (2006) Rapamycin inhibits cell motility by suppression of mTOR-mediated S6K1 and 4E-BP1 pathways. Oncogene 25(53):7029–7040 16715128 10.1038/sj.onc.1209691

[48] Heesom KJ, Denton RM (1999) Dissociation of the eukaryotic initiation factor-4E/4E-BP1 complex involves phosphorylation of 4E-BP1 by an mTOR-associated kinase. FEBS Lett 457(3):489–493 10471835 10.1016/s0014-5793(99)01094-7

[49] Carracedo A, Ma L, Teruya-Feldstein J, Rojo F, Salmena L, Alimonti A, Egia A, Sasaki AT, Thomas G, Kozma SC, Papa A, Nardella C, Cantley LC, Baselga J, Pandolfi PP (2008) Inhibition of mTORC1 leads to MAPK pathway activation through a PI3K-dependent feedback loop in human cancer. J Clin Invest 118(9):3065–3074 18725988 10.1172/JCI34739 PMC2518073

[50] Roux PP, Shahbazian D, Vu H, Holz MK, Cohen MS, Taunton J, Sonenberg N, Blenis J (2007) RAS/ERK signaling promotes site-specific ribosomal protein S6 phosphorylation via RSK and stimulates cap-dependent translation. J Biol Chem 282(19):14056–14064 17360704 10.1074/jbc.M700906200 PMC3618456

[51] Hsieh AC, Ruggero D (2010) Targeting eukaryotic translation initiation factor 4E (eIF4E) in cancer. Clin Cancer Res 16(20):4914–4920 20702611 10.1158/1078-0432.CCR-10-0433 PMC7539621

[52] Silvera D, Formenti SC, Schneider RJ (2010) Translational control in cancer. Nat Rev Cancer 10(4):254–266 20332778 10.1038/nrc2824

[53] Topisirovic I, Ruiz-Gutierrez M, Borden KL (2004) Phosphorylation of the eukaryotic translation initiation factor eIF4E contributes to its transformation and mRNA transport activities. Cancer Res 64(23):8639–8642 15574771 10.1158/0008-5472.CAN-04-2677

[54] Furic L, Rong L, Larsson O, Koumakpayi IH, Yoshida K, Brueschke A, Petroulakis E, Robichaud N, Pollak M, Gaboury LA, Pandolfi PP, Saad F, Sonenberg N (2010) eIF4E phosphorylation promotes tumorigenesis and is associated with prostate cancer progression. Proc Natl Acad Sci USA 107(32):14134–14139 20679199 10.1073/pnas.1005320107 PMC2922605

[55] Ruan H, Li X, Xu X, Leibowitz BJ, Tong J, Chen L, Ao L, Xing W, Luo J, Yu Y, Schoen RE, Sonenberg N, Lu X, Zhang L, Yu J (2020) eIF4E S209 phosphorylation licenses Myc- and stress-driven oncogenesis. Elife 9:e60151 33135632 10.7554/eLife.60151 PMC7665890

[56] Huber S, Oelsner M, Decker T, zum Buschenfelde CM, Wagner M, Lutzny G, Kuhnt T, Schmidt B, Oostendorp RA, Peschel C, Ringshausen I (2011) Sorafenib induces cell death in chronic lymphocytic leukemia by translational downregulation of Mcl-1. Leukemia 25(5):838–847 21293487 10.1038/leu.2011.2

[57] Rahmani M, Davis EM, Bauer C, Dent P, Grant S (2005) Apoptosis induced by the kinase inhibitor BAY 43–9006 in human leukemia cells involves down-regulation of Mcl-1 through inhibition of translation. J Biol Chem 280(42):35217–35227 16109713 10.1074/jbc.M506551200

[58] Lin CJ, Cencic R, Mills JR, Robert F, Pelletier J (2008) C-Myc and eIF4F are components of a feedforward loop that links transcription and translation. Cancer Res 68(13):5326–5334 18593934 10.1158/0008-5472.CAN-07-5876

[59] Waskiewicz AJ, Johnson JC, Penn B, Mahalingam M, Kimball SR, Cooper JA (1999) Phosphorylation of the cap-binding protein eukaryotic translation initiation factor 4E by protein kinase Mnk1 in vivo. Mol Cell Biol 19(3):1871–1880 10022874 10.1128/mcb.19.3.1871 PMC83980

[60] Ueda T, Watanabe-Fukunaga R, Fukuyama H, Nagata S, Fukunaga R (2004) Mnk2 and Mnk1 are essential for constitutive and inducible phosphorylation of eukaryotic initiation factor 4E but not for cell growth or development. Mol Cell Biol 24(15):6539–6549 15254222 10.1128/MCB.24.15.6539-6549.2004 PMC444855

[61] Pyronnet S (2000) Phosphorylation of the cap-binding protein eIF4E by the MAPK-activated protein kinase Mnk1. Biochem Pharmacol 60(8):1237–1243 11007962 10.1016/s0006-2952(00)00429-9

[62] Pyronnet S, Imataka H, Gingras AC, Fukunaga R, Hunter T, Sonenberg N (1999) Human eukaryotic translation initiation factor 4G (eIF4G) recruits mnk1 to phosphorylate eIF4E. EMBO J 18(1):270–279 9878069 10.1093/emboj/18.1.270 PMC1171121

[63] O’Loghlen A, González VM, Pineiro D, Pérez-Morgado MI, Salinas M, Martín ME (2004) Identification and molecular characterization of Mnk1b, a splice variant of human MAP kinase-interacting kinase Mnk1. Exp Cell Res 299(2):343–355 15350534 10.1016/j.yexcr.2004.06.006

[64] O’Loghlen A, González VM, Jurado T, Salinas M, Martín ME (2007) Characterization of the activity of human MAP kinase-interacting kinase Mnk1b. Biochim Biophys Acta 1773(9):1416–1427 17590453 10.1016/j.bbamcr.2007.05.009

[65] Broecker-Preuss M, Muller S, Britten M, Worm K, Schmid KW, Mann K, Fuhrer D (2015) Sorafenib inhibits intracellular signaling pathways and induces cell cycle arrest and cell death in thyroid carcinoma cells irrespective of histological origin or BRAF mutational status. BMC Cancer 15:184 25879531 10.1186/s12885-015-1186-0 PMC4377064

[66] Wang Q, Wu G, Che X, Li Q, Zhang Z, Tang Q (2018) Sorafenib induces renal cell carcinoma apoptosis *via* upregulating activating transcription factor 4. Pharmazie 73(3):156–160 29544563 10.1691/ph.2018.7855

[67] Contreras L, Rodriguez-Gil A, Muntané J, de la Cruz J (2025) Sorafenib-associated translation reprogramming in hepatocellular carcinoma cells. RNA Biol in the press. 10.1080/15476286.2025.2483484 PMC11934173 40116042

[68] Sonenberg N, Hinnebusch AG (2009) Regulation of translation initiation in eukaryotes: mechanisms and biological targets. Cell 136(4):731–745 19239892 10.1016/j.cell.2009.01.042 PMC3610329

[69] Matter MS, Decaens T, Andersen JB, Thorgeirsson SS (2014) Targeting the mTOR pathway in hepatocellular carcinoma: current state and future trends. J Hepatol 60(4):855–865 24308993 10.1016/j.jhep.2013.11.031 PMC3960348

[70] Villanueva A, Chiang DY, Newell P, Peix J, Thung S, Alsinet C, Tovar V, Roayaie S, Minguez B, Sole M, Battiston C, Van Laarhoven S, Fiel MI, Di Feo A, Hoshida Y, Yea S, Toffanin S, Ramos A, Martignetti JA, Mazzaferro V et al (2008) Pivotal role of mTOR signaling in hepatocellular carcinoma. Gastroenterology 135(6):1972–1983 18929564 10.1053/j.gastro.2008.08.008 PMC2678688

[71] Navarro-Villarán E, de la Cruz-Ojeda P, Contreras L, González R, Negrete M, Rodríguez-Hernández MA, Marín-Gómez LM, Álamo-Martínez JM, Calvo A, Gómez-Bravo MA, de la Cruz J, Padillo J, Muntané J (2020) Molecular pathways leading to induction of cell death and anti-proliferative properties by Tacrolimus and mTOR inhibitors in liver cancer cells. Cell Physiol Biochem 54(3):457–473 32369692 10.33594/000000230

[72] Hou J, Lam F, Proud C, Wang S (2012) Targeting Mnks for cancer therapy. Oncotarget 3(2):118–131 22392765 10.18632/oncotarget.453 PMC3326643

[73] Scheper GC, Proud CG (2002) Does phosphorylation of the cap-binding protein eIF4E play a role in translation initiation? Eur J Biochem 269(22):5350–5359 12423333 10.1046/j.1432-1033.2002.03291.x PMC7163980

[74] McKendrick L, Morley SJ, Pain VM, Jagus R, Joshi B (2001) Phosphorylation of eukaryotic initiation factor 4E (eIF4E) at Ser209 is not required for protein synthesis *in vitro* and *in vivo*. Eur J Biochem 268(20):5375–5385 11606200 10.1046/j.0014-2956.2001.02478.x

[75] Lachance PE, Miron M, Raught B, Sonenberg N, Lasko P (2002) Phosphorylation of eukaryotic translation initiation factor 4E is critical for growth. Mol Cell Biol 22(6):1656–1663 11865045 10.1128/MCB.22.6.1656-1663.2002 PMC135594

[76] Romagnoli A, D’Agostino M, Ardiccioni C, Maracci C, Motta S, La Teana A, Di Marino D (2021) Control of the eIF4E activity: structural insights and pharmacological implications. Cell Mol Life Sci 78(21–22):6869–6885 34541613 10.1007/s00018-021-03938-z PMC8558276

[77] Siddiqui N, Sonenberg N (2015) Signalling to eIF4E in cancer. Biochem Soc Trans 43(5):763–772 26517881 10.1042/BST20150126 PMC4613458

