# Supplementary material for: The many ways to inhibit translation by Sorafenib in liver cancer cells
Source: Mol Cell Biochem. 2025 Sep 15;481(1):245–62. doi: 10.1007/s11010-025-05391-z (PMC12906594; doi:10.1007/s11010-025-05391-z)
Supplement: Supplementary file 1 — Supplementary file1 (DOCX 928 kb) [file 11010_2025_5391_MOESM1_ESM.docx]

**SUPPLEMENTAL INFORMATION**

**The many ways to inhibit translation by Sorafenib in liver cancer cells**

Laura Contreras^1,2^, Sara Ricciardi^4,5^, Stefano Biffo^4,5^,, Jordi Muntané^1,3,6,*^ and Jesús de la Cruz^1,2,*^

^1^Instituto de Biomedicina de Sevilla, Hospital Universitario Virgen del Rocío/CSIC/Universidad de Sevilla, E-41013 Seville, Spain

^2^Departamento de Genética, Facultad de Biología, Universidad de Sevilla, Seville, Spain

^3^Departamento de Fisiología Médica y Biofísica, Universidad de Sevilla, Sevilla, Spain

^4^National Institute of Molecular Genetics, INGM Fondazione Romeo ed Enrica Invernizzi, Milan, Italy

^5^Department of Biosciences, University of Milan, Milan, Italy

^6^Centro de Investigación Biomédica en Red de Enfermedades Hepáticas y Digestivas (CIBEREHD), Madrid, Spain

**^*^ To whom correspondence may be addressed:**

Jordi Muntané. E-mail: jmuntane-ibis@us.es

Jesús de la Cruz. E-mail: jdlcd@us.es

**Table S1.** Primary antibodies used in this study

| **Antigen** | **Type** | **Dilution** | **Source** |
| --- | --- | --- | --- |
| Phospho-4E-BP1 (S65) | Rabbit polyclonal | 1:2000 | #9451, Cell Signaling |
| Phospho-4E-BP1 (T37/46) | Rabbit polyclonal | 1:2000 | #9459, Cell Signaling |
| 4E-BP1 | Rabbit polyclonal | 1:2000 | #9452, Cell Signaling |
| Phospho-eIF2α (S51) | Rabbit polyclonal | 1:2000 | #9721, Cell Signaling |
| eIF2α (D7D3) | Rabbit monoclonal | 1:2000 | #5324, Cell Signaling |
| Cyclin D1 (M-20) | Rabbit polyclonal | 1:1000 | Sc-718, Santa Cruz |
| c-Myc (D84C12) | Rabbit monoclonal | 1:1000 | #5605, Cell Signaling |
| c-Myc (9E10) | Mouse monoclonal | 1:1000 | Sc-40, Santa Cruz |
| eIF4A (C32B4) | Rabbit monoclonal | 1:2000 | #2013, Cell Signaling |
| eIF4G (C45A4) | Rabbit monoclonal | 1:2000 | #2469, Cell Signaling |
| Phospho-ERK1/2 (T202/Y204) (D13.14.4E) | Rabbit monoclonal | 1:2000 | #4370, Cell Signaling |
| ERK1/2 | Rabbit polyclonal | 1:2000 | #9102, Cell Signaling |
| Phospho-eIF4E (S209) | Rabbit polyclonal | 1:4000 | #9741, Cell Signaling |
| eIF4E | Rabbit polyclonal | 1:2000 | #9742, Cell Signaling |
| Phospho-RPS6 (S235/236) | Rabbit polyclonal | 1:5000 | #2211, Cell Signaling |
| Phospho-RPS6 (S240/244) (D68F8) | Rabbit monoclonal | 1:5000 | #5364, Cell Signaling |
| RPS6 (5G10) | Rabbit monoclonal | 1:5000 | #2217, Cell Signaling |
| GAPDH (0411) | Mouse monoclonal | 1:1000 | Sc-47724, Santa Cruz |
| Vinculin (H-10) | Mouse monoclonal | 1:1000 | Sc-25336, Santa Cruz |
| Flag (M2) | Mouse monoclonal | 1:1000 | F3165, MilliporeSigma |
| Puromycin (12D10) | Mouse monoclonal | 1:10,000 | MABE343, MilliporeSigma |
| β-actin (mAbcam 8224) | Mouse monoclonal | 1:1000 | ab8224, Abcam |
| α-tubulin (EP1332Y) | Rabbit monoclonal | 1:1000 | ab52866, Abcam |

**Table S2.** Sequences of shRNAs and oligonucleotides used for sequencing

| **Oligonucleotide** | **Forward (5'-3')** | **Reverse (5'-3')** |
| --- | --- | --- |
| 4E-BP1-shRNA | CCGGCGGTGAAGAGTCACAGTTTGACTCGAGTCAAACTGTGACTCTTCACCGTTTTTG | AATTCAAAAACGGTGAAGAGTCACAGTTTGACTCGAGTCAAACTGTGACTCTTCACCG |
| 4E-BP2-shRNA | CCGGGCTGTATTTCTGTAGAGCTAACTCGAG  TTAGCTCTACAGAAATACAGCTTTTTG | AATTCAAAAAGCTGTATTTCTGTAGAGCTAA  CTCGAGTTAGCTCTACAGAAATACAGC |
| PLKO.1 | CAAGGCTGTTAGAGAGATAATTGGA |  |

**Table S3.** Primers used for RT-qPCR

| **Primer** | **Forward (5'-3')** | **Reverse (5'-3')** |
| --- | --- | --- |
| Cyclin D1 | GTGAAGTTCATTTCCAATCCG | GGTCACACTTGATCA CTC TGG |
| c-Myc | GGAGGAGACATGGTGAACCAG | AGGAGGCCAGCTTCTCTGAGA |
| Mcl-1 | GCTTCAGTCTCGGAACATGAC | CTTATGGCTCTGAGATGGGC |
| VEGFA | CCATCCAATCGAGACCCTGG | CTCCAGGCCCTCGTCATTG |
| RPS6 | TGTCCGCCTGCTACTGAGTAA | GCAACCACGAACTGATTTTCTC |
| RPS18 | GCGGGAGAACTCACTGAGG | CGTGGATTCTGCATAATGGTGAT |
| RPL32 | GCCCAAGATCGTCAAAAAGAGA | TCCGCCAGTTACGCTTAATTT |
| ATF4 | CACTAGGTACCGCCAGAAGA | AATCCGCCCTCTCTTTTAGA |
| β-actin | TCCCTGGAGAAGAGCTACGA | AGGAAGGAAGGCTGGAAGAG |
| 28S rRNA | CAAAGCGGGTGGTAAACTCC | TTCACGCCCTCTTGAACTCT |
| Luciferase | ATCCGGAAGCGACCAACGCC | GTCGGGAAGACCTGCCACGC |


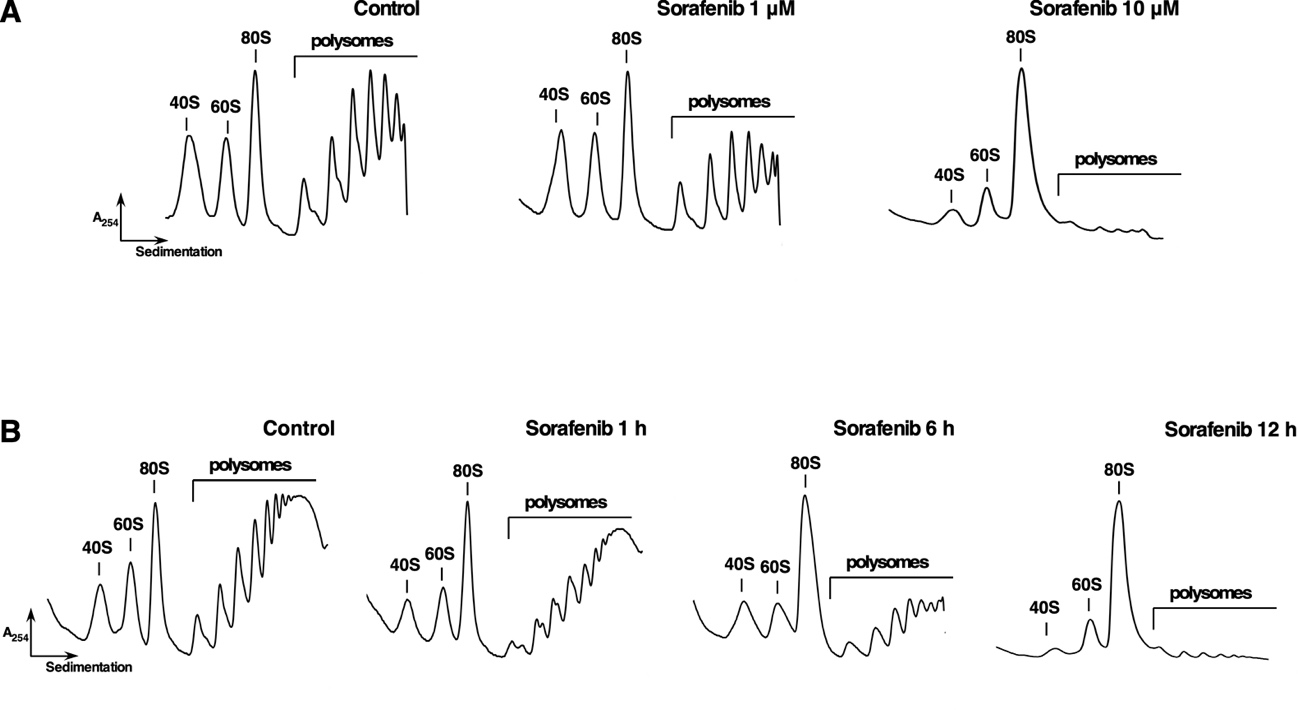


**Fig. S1.** Inhibition of translation by Sorafenib. (**A**) Dose-dependent inhibition of translation by Sorafenib. HepG2 cells were mock-treated (Control) or treated with 1 μM or 10 μM Sorafenib for 12 h, and subjected to polysome profile analysis. (**B**) Time-dependent inhibition of translation by Sorafenib. HepG2 cells were mock-treated (Control) or treated with 10 μM Sorafenib for 1, 6, and 12 h, and subjected to polysome profile analysis. In all circumstances, whole cell extracts were prepared following the procedure described in the Materials and Methods section. Ten A_260_ units of each extract were resolved in 7 to 50% sucrose gradients. The A_254_ was continuously monitored. Sedimentation is from left to right. The identity of the different peaks is indicated. Experiments were repeated three times; representative profiles are shown.


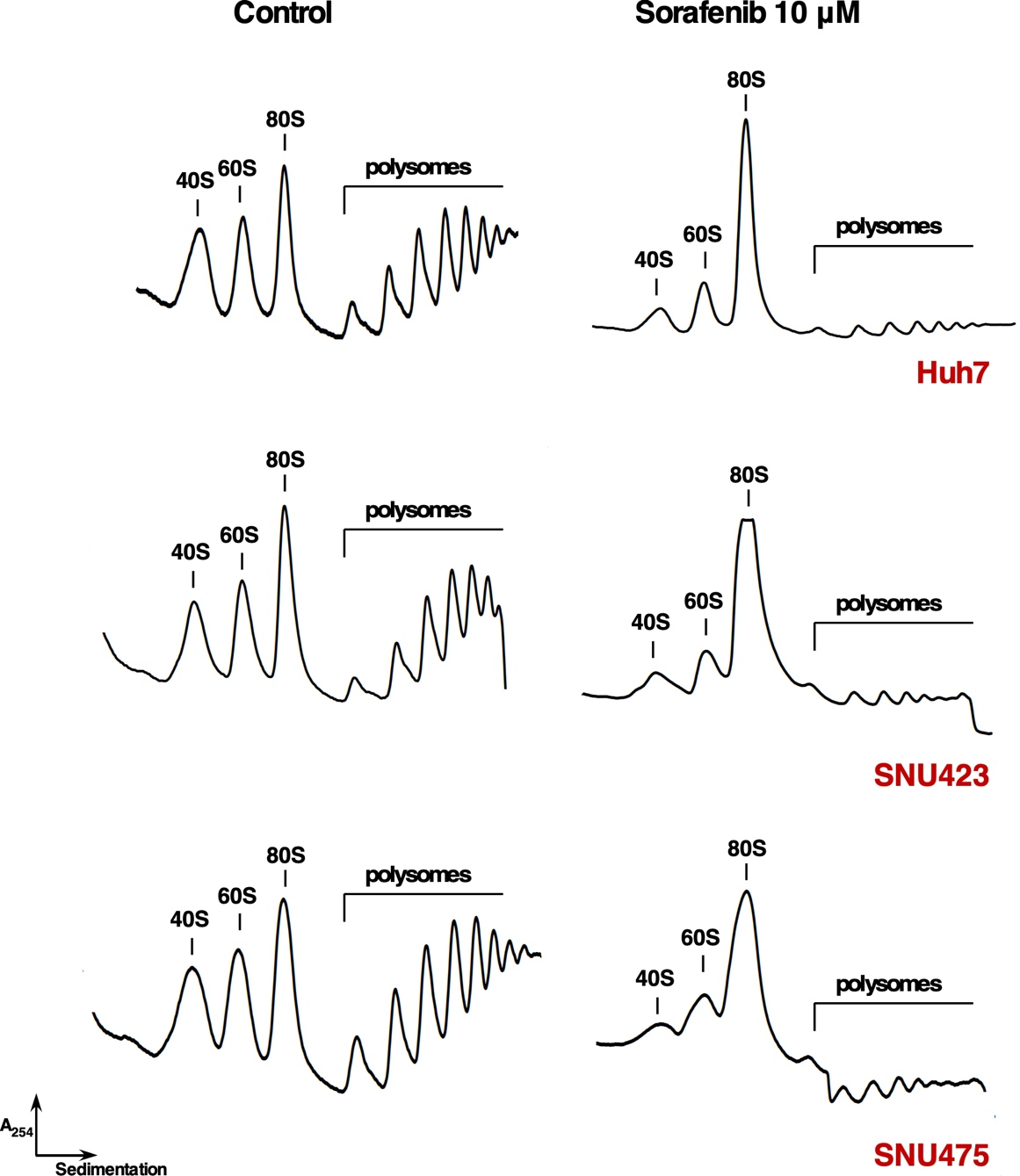


**Fig. S2.** Sorafenib leads to translation inhibition in different HCC cell lines. Polysome profiles were obtained from Huh7 (obtained from Apath), SNU423 (CRL-2238, obtained from ATCC/LGC Standards) and SNU475 (CRL-2236, obtained from ATCC/LGC Standards) cells, which were mock-treated (Control) or treated with 10 μM Sorafenib for 12 h. Cell extracts and polysome profile analysis were performed following the procedure described in Material and Methods. Ten A_260_ units of each extract were resolved in 7 to 50% sucrose gradients. The A_254_ was continuously monitored. Sedimentation is from left to right. The identity of the different peaks is indicated. Experiments were repeated three times; representative profiles are shown.


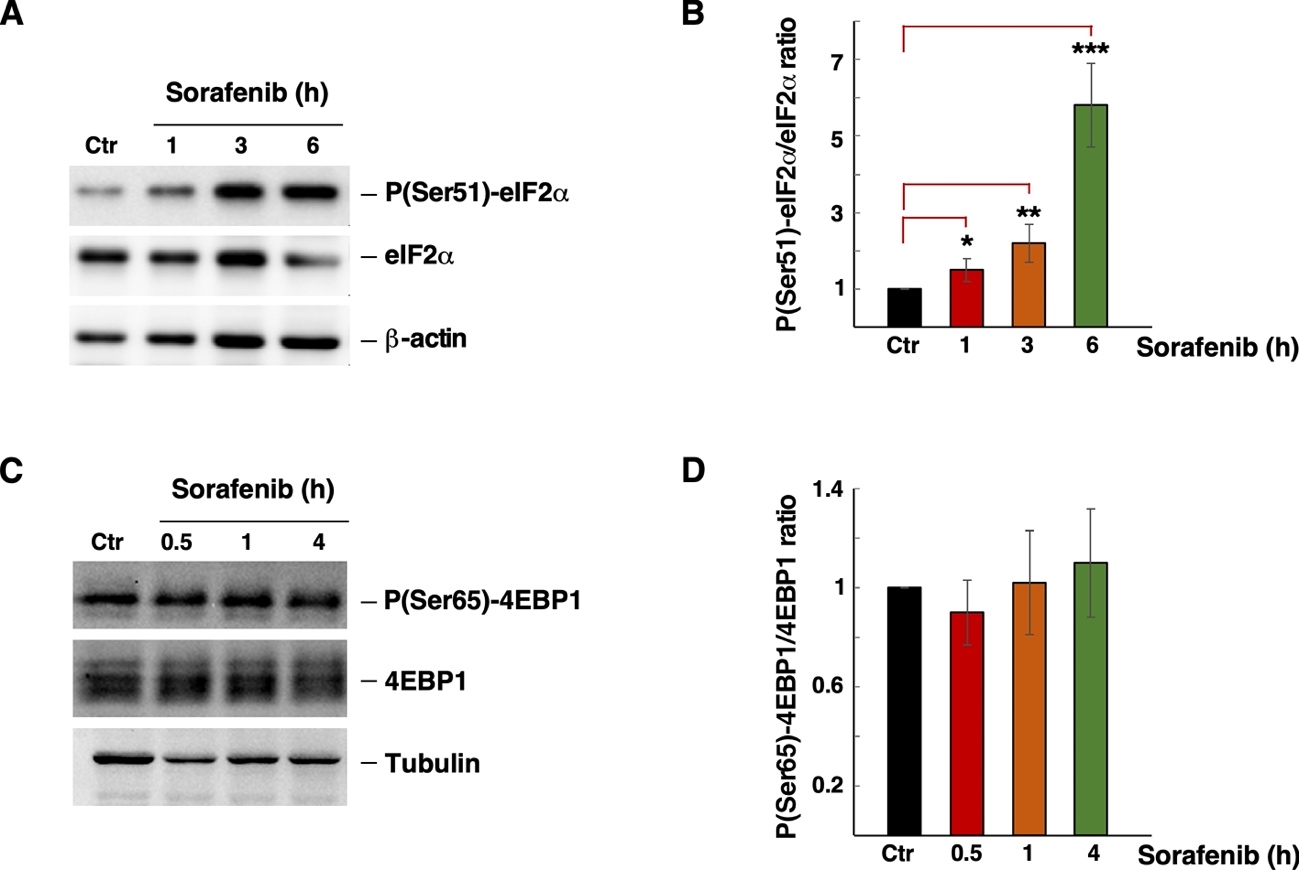


**Fig. S3.** Role of the phosphorylation status of eIF2α and 4E-BPs in the translation inhibition exerted by Sorafenib. Kinetics of eIF2α and 4E-BP1 phosphorylation. Total protein extracts were obtained from mock-treated (Ctr) and cells treated with 10 μM Sorafenib for the indicated times. Extracts were then analysed by western blotting with antibodies directed against the total and Ser51-phosphorylated forms of eIF2α, and β-actin (**A**), or against the total and Ser65-phosphorylated forms of 4E-BP1, and α-tubulin (**C**), respectively. (**B** and **D**) Densitometric analysis: the phospho-eIF2α/total eIF2α ratio relative to the loading control (β-actin) and of the phospho-4E-BP1/total 4E-BP1 ratio relative to the loading control (α-tubulin) were calculated and normalized to that of the control, which was set arbitrarily at 1.0. Three independent experiments were performed, and data are expressed as means ± SDs. Statistical significance was analysed by Student's *t*-test (* *p* < 0.05; ** *p* < 0.01; *** *p* < 0.001). Note that the analyses provide a non-significant difference between the means of the 4E-BP1 ratios.


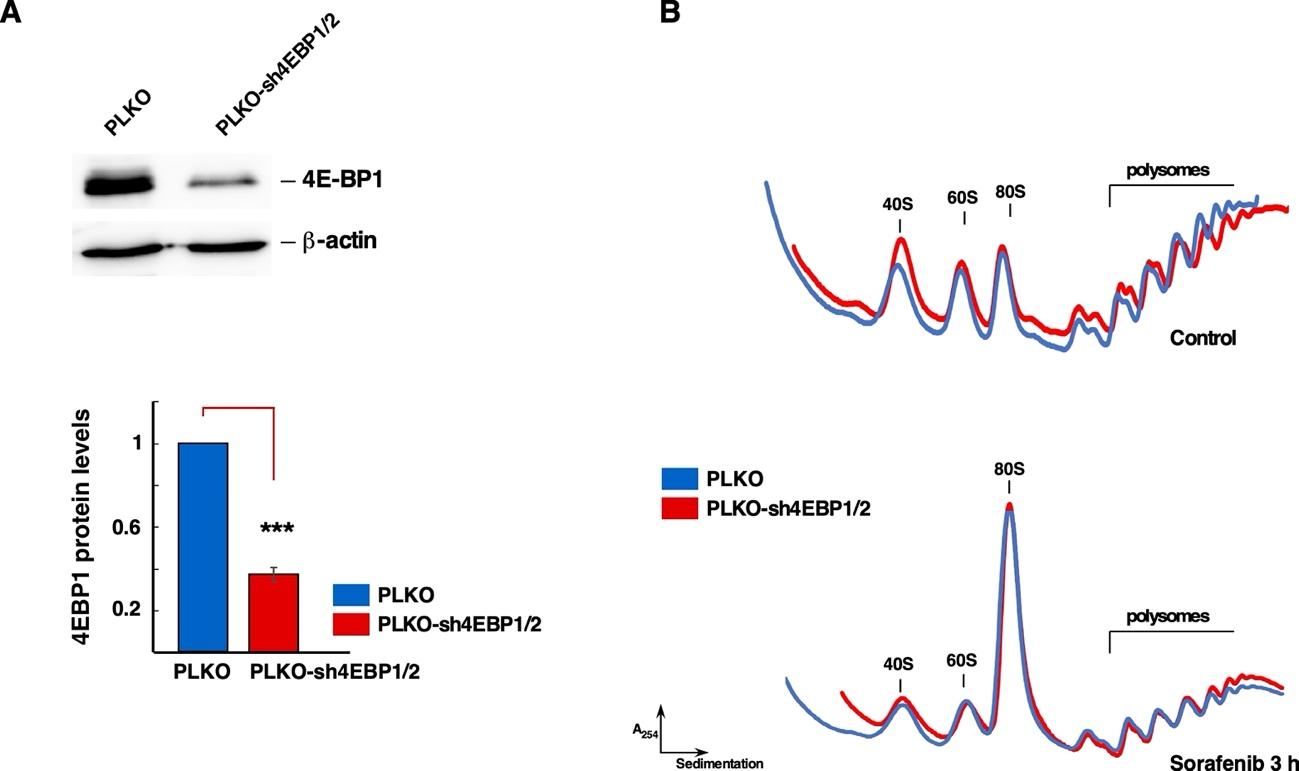


**Fig. S4.** Silencing of 4E-BP1 does not restore the translation inhibition exerted by Sorafenib. (**A**) Effect on 4E-BP1 protein levels after silencing expression of 4E-BP1/2. Total protein extracts from 4E-BP1/2-silenced (PLKO-4E-BP1/2) and control (PLKO) cells treated with 10 μM Sorafenib for 3 h were analysed by western blotting with specific antibodies against 4E-BP1 and β-actin. Upon densitometric analysis, the 4E-BP1 protein levels were calculated and normalized to that of the control, which was set arbitrarily at 1.0. Three independent experiments were performed and data are expressed as means ± SDs. Statistical significance was analysed by Student's *t*-test (*** *p* < 0.001). (**B**) The inhibition of translation by Sorafenib is not modified upon 4E-BP-silencing. Translation was checked by polysome profile analysis in the above cells under the same conditions. Ten A_260_ units of each extract were resolved in 7 to 50% sucrose gradients. The A_254_ was continuously monitored. Sedimentation is from left to right. The identity of the different peaks is indicated. Representative profiles are shown.

**
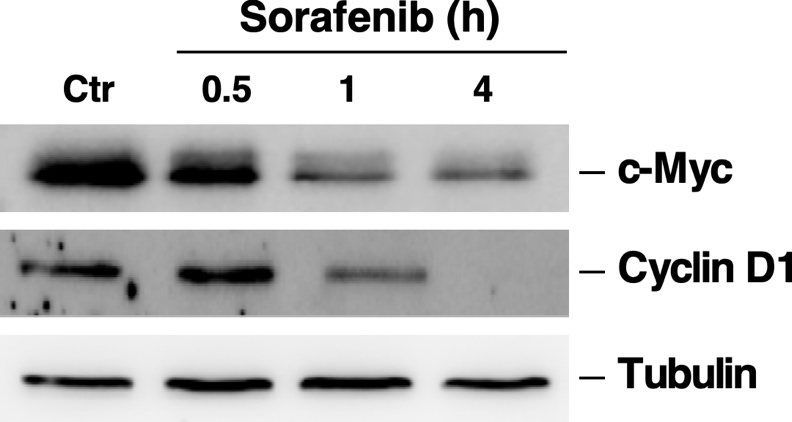
**

**Fig. S5.** Time-course of eIF4E-target proteins levels upon a Sorafenib treatment. Total protein extracts from HepG2 cells untreated (Ctr) or treated with 10 μM Sorafenib at the indicated times were obtained and analysed by western blotting as described in the Materials and Methods section. Tubulin was used as loading control. The signals of c-Myc, Cyclin D1, and Tubulin were detected using specific antibodies. Representative blots are shown.


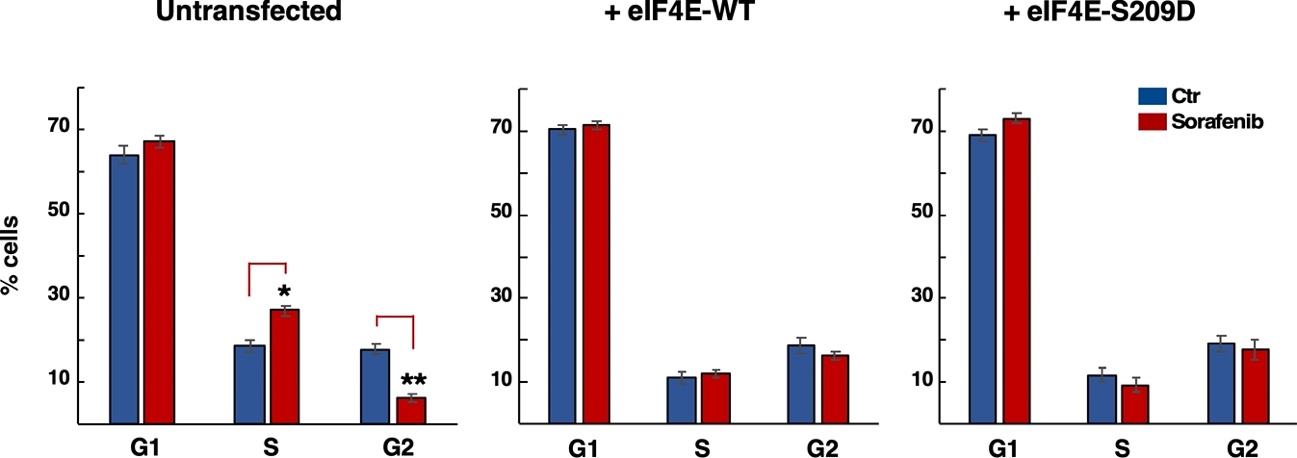


**Fig. S6.** Sorafenib does not induce a cell cycle arrest in cells overexpressing either eIF4E-WT or eIF4E-S209D. Histograms of the percentage of cells in each cell cycle phase based on three independent experiments. Cells were untransfected or transfected with pLPCX-eIF4E-WT and pLPCX-eIF4E-S209D for 48 h. Cell extracts from untreated (Ctr) or 10 μM Sorafenib-treated cells for 12 h were obtained and analysed by flow cytometry as described in the Materials and Methods section. The percentage of cells at the different phases is shown in the histogram. Results are expressed as means ± SDs. Statistical significance was analysed by Student’s *t*-test (* *p* < 0.05; ** *p* < 0.01). Note that the analysis of the cell cycle stages of untransfected cells have been previously reported [1].


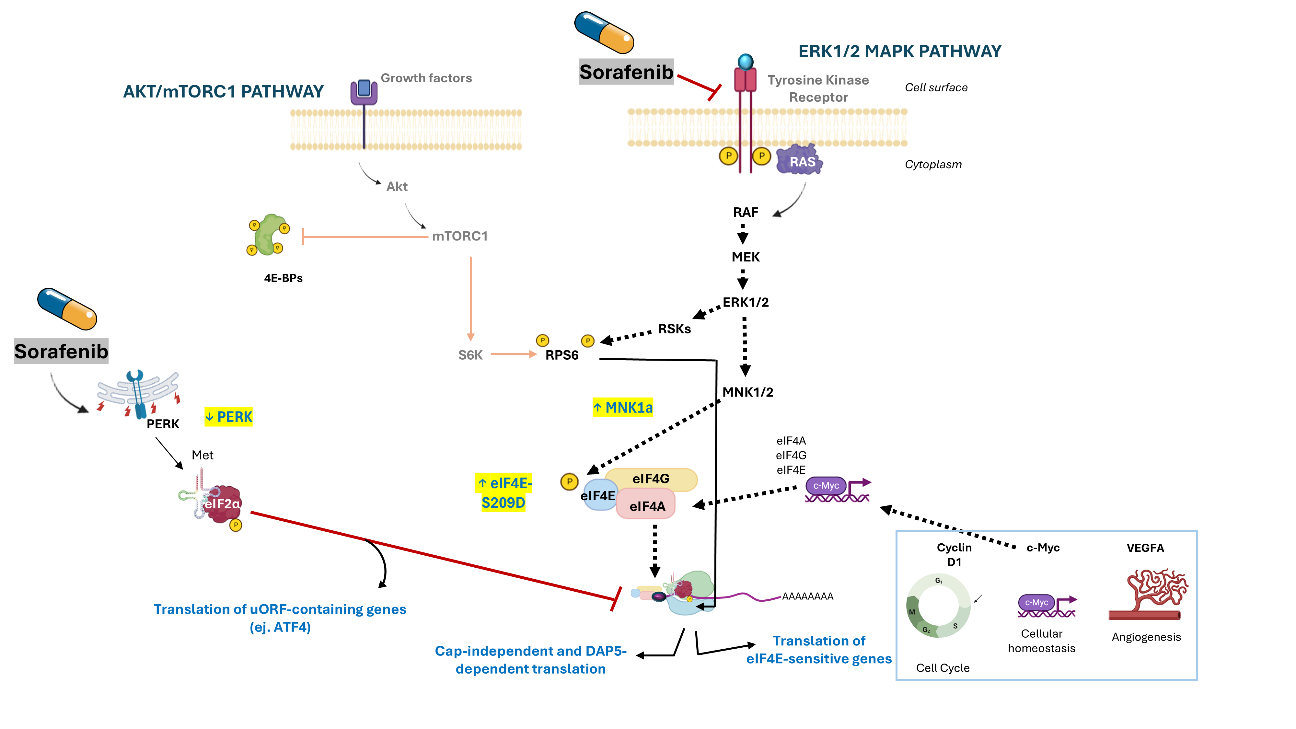


**Fig. S7.** Sorafenib hierarchically affects different signalling pathways controlling translation, which leads to translation reprogramming. This figure summarises our results and depicts the signalling pathways whose signals converge into the translational apparatus and are dysregulate in Sorafenib-treated HepG2 cells. Our results clearly show that (i) the downregulation of MAPKs causes a reduction in the phosphorylation levels of RPS6 at its Ser-235 and Ser-236, and eIF4E at its Ser-209, resulting in the selective translation inhibition of a subset of pro-tumoral mRNAs involved in cell cycle progression (e.g., Cyclin D1), cellular homeostasis (e.g., c-Myc), and angiogenesis (e.g., VEGFA). (ii) Sorafenib also induces ER stress, triggering the UPR. Thus, PERK-induced phosphorylation of eIF2α inhibits overall translation and stimulates translation of a subset of mRNAs containing upstream ORFs, such as the transcriptional factor ATF4, a regulator of stress response in human cells. (iii) Sorafenib also negatively affect the accumulation of eIF4A and eIF4G, which disrupts the assembly of eIF4F complex and likely compromises the translation of cap-dependent genes, favouring that of cap-independent and DAP5-dependent ones. We hypothesise that the latter is the result of the action of Sorafenib on the feedforward loop that links c-Myc with the cap-dependent complex. Some of our conclusions derives from the results obtained upon silencing PERK, overexpression of MNK1a, and overexpression of either eIF4E-WT or the phosphomimetic eIF4E-S209D isoform (fluorescent yellow highlighter traces).

**SUPPLEMENTAL REFERENCES**

1. Contreras L, Rodríguez-Gil A, Muntané J, de la Cruz J (2022) Broad transcriptomic impact of Sorafenib and its relation to the antitumoral properties in liver cancer cells. Cancers (Basel) 14(5).
